# Whole-genome prediction of bacterial pathogenic capacity on novel bacteria using protein language models with PathogenFinder2

**DOI:** 10.1093/bioinformatics/btag129

**Published:** 2026-05-28

**Authors:** Alfred Ferrer Florensa, Jose Juan Almagro Armenteros, Rolf Sommer Kaas, Philip Thomas Lanken Conradsen Clausen, Henrik Nielsen, Burkhard Rost, Frank Møller Aarestrup

**Affiliations:** Research Group for Genomic Epidemiology, National Food Institute, Technical University of Denmark, Kongens Lyngby, 2800, Denmark; Informatics and Predictive Sciences Research, Bristol Myers Squibb Company, Sevilla, 41092, Spain; Research Group for Genomic Epidemiology, National Food Institute, Technical University of Denmark, Kongens Lyngby, 2800, Denmark; Research Group for Genomic Epidemiology, National Food Institute, Technical University of Denmark, Kongens Lyngby, 2800, Denmark; Bioinformatics, Department of Health Technology, Technical University of Denmark, Kongens Lyngby, 2800, Denmark; Rostlab, Department of Bioinformatics and Computational Biology, Technical University of Munich, Munich, 85748, Germany; Research Group for Genomic Epidemiology, National Food Institute, Technical University of Denmark, Kongens Lyngby, 2800, Denmark

## Abstract

**Motivation:**

Infectious diseases continue to be a leading cause of mortality and pose a significant global health threat. Thus, the development of tools for surveillance and early detection of emerging pathogens is needed.

**Results:**

We introduce PathogenFinder2, a novel, alignment-free, taxonomy-agnostic model for predicting bacterial pathogenic capacity in humans using protein language models. It outperforms previous methods, particularly for novel taxa, and provides interpretable outputs by highlighting proteins most relevant to pathogenic potential. These insights aid the identification of virulence factors, vaccine targets, and infection-related metabolic pathways. Furthermore, we introduce the Bacterial Pathogenic Capacity Landscape, which reveals patterns linked to host condition, infection site, microbial antagonism, and environmental origin.

**Availability:**

The model is freely available online at https://genepi.dk/pathogenfinder2, or as a standalone program (https://github.com/genomicepidemiology/PathogenFinder2).

## 1 Introduction

Human-bacterial interactions vary significantly. While some bacteria cannot colonize humans, others engage in non-damaging or even beneficial interactions like mutualism and commensalism, or harmful ones as pathogens (Casadevall and Pirofski 2000, Dethlefsen *et al*. 2007). New possible beneficial bacteria are today widely explored for health and commercial purposes, including starter cultures, pre- and probiotics, and industrial applications. Moreover, with anticipated climate changes and greater exploration of natural resources, human exposure to novel bacterial taxa are also expected to rise. Therefore, it is crucial to develop tools to distinguish harmful bacteria from harmless ones, enabling early detection and timely interventions, even when faced with novel taxa.

Differentiating between pathogenic and non-pathogenic bacteria has been a longstanding challenge for the scientific community, even since Koch’s postulates (Evans 1993). The outcome of bacterial contact with humans varies significantly, as evidenced by the ongoing HIV/AIDS epidemic. Infections by bacteria previously deemed to be non-pathogenic in immunocompromised hosts challenged the binary view of pathogenicity, leading to the concept of “opportunistic pathogens” (Armstrong 1993). However, defining this term remains complex, as infection incidence of any type of pathogen is notably higher in immunocompromised hosts; and even individuals with a healthy immune system can be infected by “opportunistic pathogens” (Casadevall and Pirofski 2003, Méthot and Alizon 2014). This lack of consensus underscores the host’s role in the pathogen’s success (and its resulting damage). Factors such as the immune system, infection site, and host microbiome will significantly influence the interaction outcome (Casadevall and Pirofski 2009, Méthot and Alizon 2014).

However, the *pathogenic capacity* to cause host damage under appropriate conditions (Casadevall and Pirofski 2002) must be encoded in the bacterium’s genetic material (Wilson *et al*. 2002). Over many years, significant efforts have identified proteins and genes that define the pathogenic capacity and virulence of a bacterium. The extensive availability of bacterial genome data has significantly broadened our understanding of these virulence factors (Liu *et al*. 2022). However, many of these genes are also present in commensal bacteria (Holden *et al*. 2004), impeding their use for prediction of bacterial pathogenic capacity.

Traditionally, predicting bacterial pathogenic capacity has often involved inoculating pathogens in animal models. However, varying host ranges among bacteria limit this method’s effectiveness (Evans 1993). Another approach is to infer pathogenic capacity from the phenotype of closely related bacteria. This method’s effectiveness is constrained by database completeness, bias, and identification accuracy. Moreover, pathogenic capacity and taxonomy do not always correlate, as some species have both pathogenic and non-pathogenic strains (National Research Council (US) Committee on Scientific Milestones for the Development of a Gene Sequence-Based Classification System for the Oversight of Select Agents 2010). In fact, the acquisition of a mobile genetic element or a few point mutations can enable a bacterium to cause damage in a human host (Schmidt and Hensel 2004).

Recent methods to predict pathogenic capacity (although described as pathogenicity in their respective articles) have relied on machine learning, falling into two categories: protein- and read-based methods. Protein-based methods typically involve three steps: protein prediction, mapping to a protein family database, and prediction based on the hits identified. Current state-of-the-art solutions, such as PathogenFinder (Cosentino *et al*. 2013), BacPacs (Barash *et al*. 2019) and WSPC (Naor-Hoffmann *et al*. 2022), employ various strategies for database construction and prediction, to predict pathogenic capacity of bacterial isolates and identify key proteins involved in pathogenic capacity. Limitations of those methods include the need for assembled genomes, the exclusion of non-proteomic genetic material, and the reliance on protein alignment, which is constrained by database completeness and alignment analysis. In contrast, read-based methods, such as PaPrBag (Deneke *et al*. 2017) and DeePac (Bartoszewicz *et al*. 2020), do not require genome assembly or protein prediction, nor do they necessitate mapping or the creation of a protein family database. Drawbacks for such read-based methods include the inability to explain the mechanisms underlying pathogenic capacity when modeling bacterial sequences.

Here, we introduce PathogenFinder2, a novel protein-based method for predicting bacterial pathogenic capacity using protein Language Models (pLMs), without relying on traditional alignment or mapping techniques. Unlike other phenotypes, pathogenic capacity requires a diverse array of proteins that form an intricate genome-wide pattern in bacteria. To capture this complexity, our model considers the entire set of proteins predicted from a genome to make its prediction. By representing bacteria as the ensemble of their embedded proteins using ProtT5 (Elnaggar *et al*. 2022), we leverage the extensive knowledge from pLMs, and achieve representations that vary at the strain level. These, of varying length depending on the predicted protein content of each bacterium, are the input for a deep neural network, able to: (1) report the pathogenic capacity of the isolate, with state-of-the-art performance, (2) rank each predicted protein as per importance for that prediction, (3) place the bacterial genome on the *Bacterial Pathogenic Capacity Landscape*.

## 2 Methods

### 2.1 Data collection and datasets

#### 2.1.1 PathogenFinder2 dataset: development and Test-NovelSpecies

**Figure 1:**
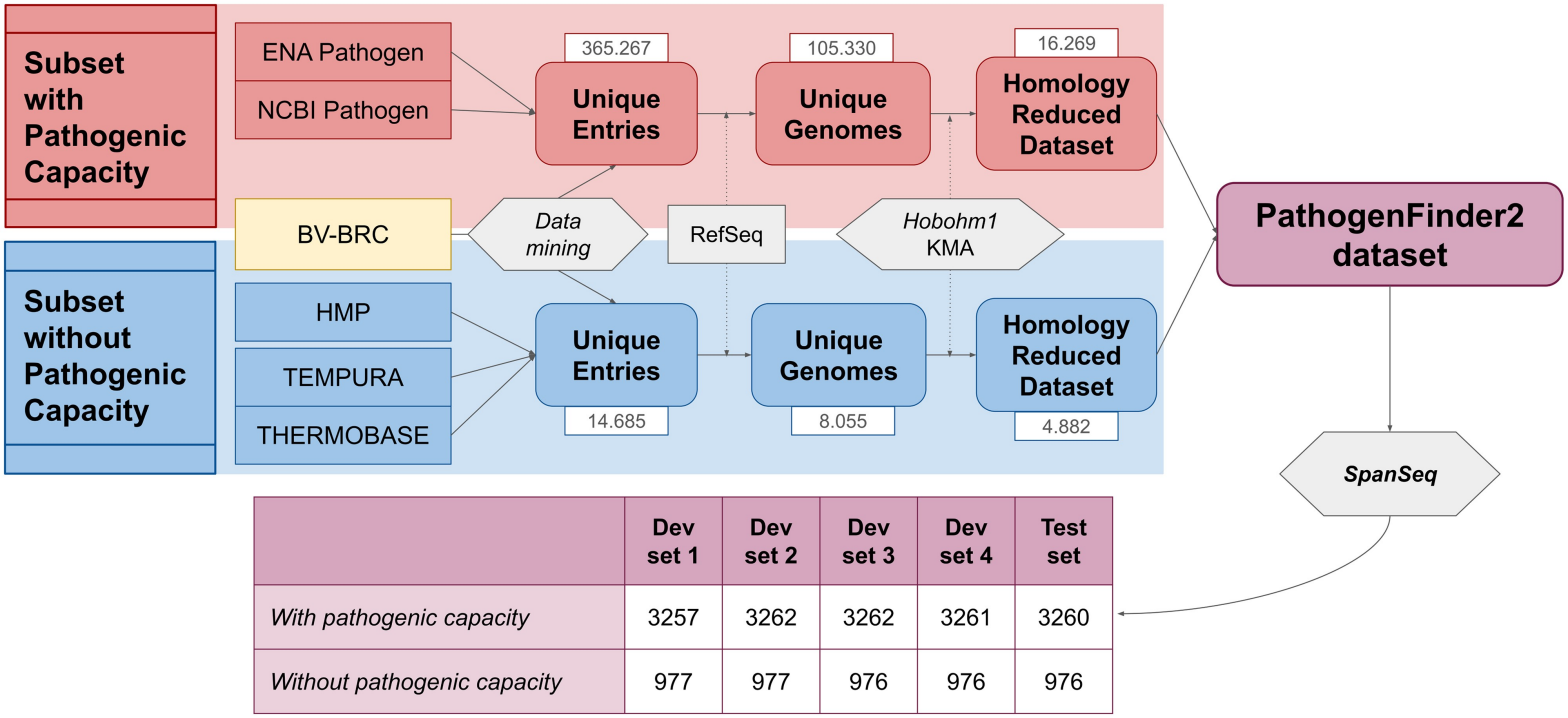
Overall scheme of the procedure to create PathogenFinder2 dataset. The scheme includes the amount of entries/genomes at each step, as well as the number of genomes with and without pathogenic capacity after homology splitting with SpanSeq. The development sets were used for hyperparameter optimization and training of the model. The numbers indicate the amount of entries/genomes at each step.

To develop and test PathogenFinder2, we compiled the largest bacterial pathogenic capacity-annotated dataset to date, using genomic sequences from publicly available databases. The dataset creation involved three major steps (Fig. 1): (1) Collection of genomic entries annotated for pathogenic capacity; (2) Linking entries to genomic sequence repositories; (3) Homology reduction and splitting. The *pathogenic* and *non-pathogenic capacity* subsets of the dataset were built separately, so a representative of similar bacterial genomes was selected only if they had the same phenotype. This was done to account for species that have strains both with and without pathogenic capacity.

For the pathogenic genomes, any bacterial sequence isolated from an infection site was included, regardless of virulence or incidence of infection on humans. The non-pathogenic genomes had to meet all following three criteria: (1) not reported as pathogenic; (2) not isolated from a sick human; (3) defined as non-pathogenic. We defined four bacterial phenotypes that could fulfill the last requirement: (a) explicitly non-pathogenic bacteria; (b) bacteria of the human microbiome; (c) bacteria classified as probiotics or used in ingested products; (d) extremophiles from environments not found in the human body. Thus, only strains categorically non-pathogenic or unable to live in the human body, or those repeatedly in contact with humans without causing disease, were selected. The final PathogenFinder2 dataset comprised 16.297 pathogenic and 4.882 non-pathogenic genomes.

For the pathogenic bacteria subset, data were collected from two databases: NCBIPathogen (Sayers *et al*. 2025) (https://www.ncbi.nlm.nih.gov/pathogens/, accessed October 2023) and ENAPathogen (Burgin *et al*. 2023) (https://www.pathogensportal.org, accessed October 2023). For NCBIPathogen, entries with human hosts from the Isolates Browser were selected. For ENAPathogen, bacterial assemblies with human hosts from the Pathogen Sequences category were downloaded using ENA Advanced Search.

To maximize data collection, the general annotated bacteria database BV-BRC (accessed October 2023, https://www.bv-brc.org/) was also used. The database was filtered for keywords indicating pathogenic capacity, improving upon previous methods with data mining techniques. Six columns in BV-BRC described disease-causing bacterial genomes: “Isolation,” “Source,” “Host Health,” “Other Clinical,” “Comments” and “Additional Metadata.” The keyword extraction process involved:

1. Tokenizing text in columns of interest
2. Removing common English words (stopwords)
3. Converting to lowercase and removing numeric/non-alphabetic tokens
4. Creating a unique token collection
5. Manually classifying tokens related to the desired phenotype (excluding species names)
6. Create a unique stem collection from tokens

The manually selected vocabulary for pathogenic bacterial cases fell into four categories (Table 1): Pathogenic, Pathogenic-related, and Disease (1 & 2).

**Table 1:** Keywords for data mining of BV-BRC.

Category	Examples (stems)	Used in
Pathogenic	pathogen-, virulen-, infec-, superbug-, …	Pathogenic Subset
Pathogenic related	fatal-, borne-, epidemi-, damag-, toxi-, outbreak-, endemi-, …	Pathogenic Subset
Disease (1)	necrotis-, bacteremi-, pneumoni-, botulism-, diphtery-, …	Pathogenic Subset
Disease (2)	gangrene-, diarrheal-, polyp-, encephalitis-, larygnitis-, …	Pathogenic Subset
Unhealthy	accident, amputation, obstruction, subacute, bleed-, …	–
Non Pathogenic	non-patho-, volunteer-, commensal-, reference-, donor-, …	Non-Pathogenic Subset
Microbiome	flora, microbiom-, gut-, inhabit-, microbiot-, …	Non-Pathogenic Subset
Probiotic	probiotic, novel food, cheese, kefir, fermented, wine, …	Non-Pathogenic Subset
Extremophile	hyperpiezophile, piezophile, termophile, cryophile, …	Non-Pathogenic Subset

Categories “Pathogenic, Pathogenic-related,” “Disease” and “Unhealthy” were created using the method of keyword extraction. Categories “Non pathogenic,” “Probiotic,” and “Extremophile” were created manually. The section “Unhealthy” is not used as a basis for creating any subset of the database, but to control that the entries of the non-pathogenic subset were coming from healthy humans (if not from probiotics or extremophiles).

After creating the vocabulary, the same database was searched for bacteria with reported cases of human harm. Initially, only entries without contamination, from the bacterial superkingdom, and with a human host were selected. Subsequently, the stems of the extracted vocabulary were searched within the specified columns of the remaining entries. Matches preceded by a negative term (e.g., “no,” “un,” or “vaccine”) or as part of an organization name (e.g., “Center for Genomic Epidemiology” which contains the stem “epidemi”) were discarded. A schematic of the entire process is shown in Supplementary Material 1, Fig. 1, available as supplementary data at *Bioinformatics* online.

To create the subset of bacteria without pathogenic capacity, data were sourced from non-pathogenic specific databases and the general BV-BRC database. For selecting bacterial extremophiles requiring conditions not present in human tissues, the entire Thermobase (DiGiacomo *et al*. 2022) database and a subset of the Tempura (Sato *et al*. 2020) database were used. Additionally, the first phase Human Microbiome Project (Turnbaugh *et al*. 2007) database was downloaded, as bacteria from healthy human microbiomes were considered mostly non-pathogenic. In BV-BRC, a similar process for selecting pathogenic entries was followed to filter non-pathogenic bacteria. However, instead of keyword extraction, a manually curated dictionary was created with categories: Non-pathogenic, Microbiome, Probiotic, and Extremophile (Table 1). The previous method for selecting entries of interest (without pathogenic capacity) was then applied, as shown in Supplementary Material 1, Fig. 1, available as supplementary data at *Bioinformatics* online. For “Probiotic” or “Extremophile” categories, bacteria did not need to have human hosts. An additional step was included to discard entries indicating unhealthy hosts or spoiled human-consumed products (Table 1, *Unhealthy*).

Entries from various databases required a RefSeq (O’Leary *et al*. 2016) or GenBank (Sayers *et al*. 2020) ID. Duplicates based on these IDs were removed, and if an ID had two different phenotypes, the non-pathogenic entries were discarded. The two subsets of genomes (with and without pathogenic capacity) were homology reduced separately using the Hobohm algorithm (Hobohm *et al*. 1992) in KMA (Clausen *et al*. 2018), not allowing for sequences in the database with a higher query coverage (in *k*-mer space) than 90%, obtaining the non-redundant dataset of genomes.

As the prediction of PathogenFinder2 is based on whole-genome information, the method for splitting the database had to consider the homology between genomes. For this, we used SpanSeq (Ferrer Florensa *et al*. 2024), to prevent data leakage between training and evaluation sets when splitting the dataset. SpanSeq calculated the *k*-mer cosine distances between all genomic sequences (*k*-mer size 18, minimizer size 16), and was set to force any pair of sequences sharing more than 70% of *k*-mers to be forced to go in the same set (Fig. 1). This threshold was chosen in order to ensure genomes from the same species appeared in the same set, as shown in (Ferrer Florensa *et al*. 2024), and allows us to evaluate our model in novel bacterial species. This produced the development set (4/5 of the data) and the *Test-NovelSpecies* (1/5 of the data). The split was also stratified to ensure the minority label (without pathogenic capacity) was represented in all sets.

#### 2.1.2 PathogenFinder2 test-2024Strains

The dataset with new bacterial entries (*Test-2024Strains*) was created following the same procedure as the original dataset (Fig. 1). Only entries submitted during 2024 were considered. Due to using the same vocabulary dictionaries from the original dataset, a manual review of the BV-BRC entries was conducted, resulting in the deletion of 15 entries due to unclear pathogenic capacity. Notice that, contrary to *Test-NewSpecies*, no homology partition strategy was used when creating this dataset, which might include species seen during training.

#### 2.1.3 Other datasets: practical cases

To assess the performance and practical utility of PathogenFinder2, we applied it to two published datasets comprising bacterial genomes from human and environmental sources. The first included *Escherichia coli*, ubiquitous bacteria with variable pathogenicity, isolates from healthy individuals (142 genomes) and patients exhibiting symptoms (152 genomes) of *E. coli* infection (Hazen *et al*. 2023). The second dataset contained 2.741 metagenome-assembled genomes (MAGs) from globally collected sewage samples, representing a typical use case for pathogen surveillance (Jespersen *et al*. 2023).

### 2.2 PathogenFinder2 architecture

**Figure 2:**
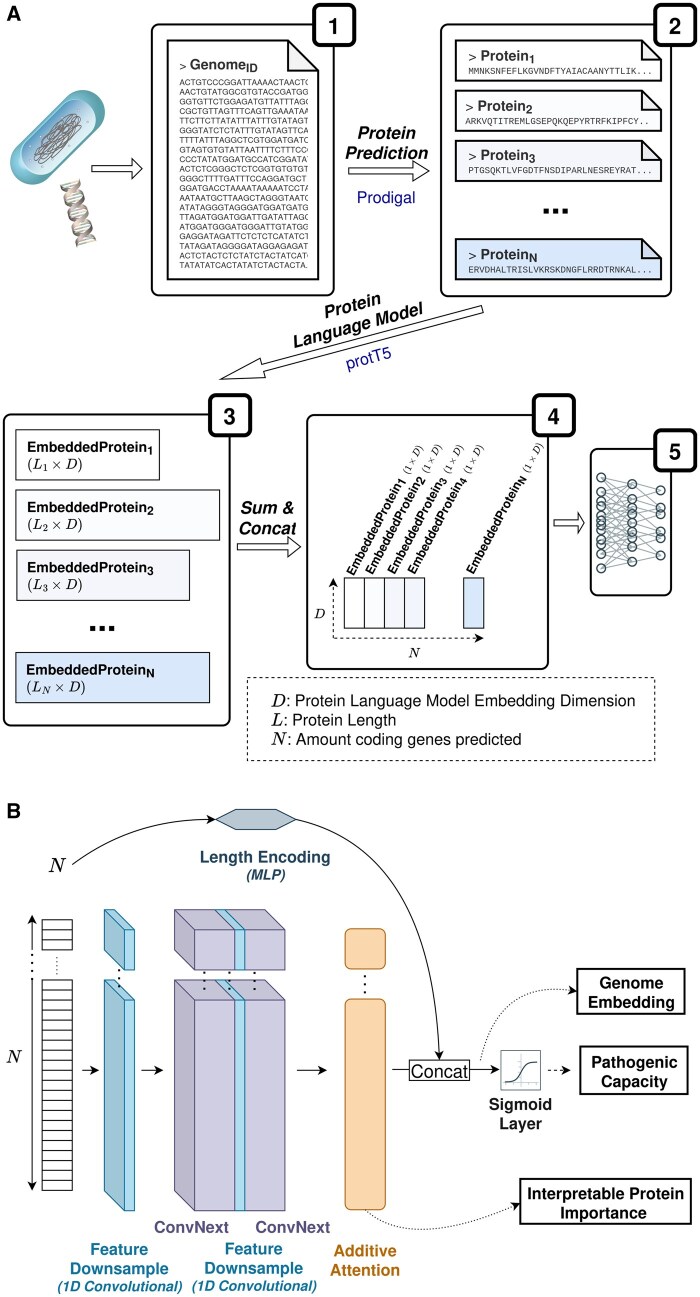
The PathogenFinder2 model. (A) Overall pipeline of PathogenFinder2, from the assembled genome until the neural network. The genome is converted into a collection of predicted proteins using Prodigal (Hyatt *et al.* 2010), which are then embedded using the protT5 (Elnaggar *et al.* 2022) protein language model. The embeddings are summed along the amino acid length axis, resulting in a 1xD vector for each protein (D being the dimensions of the protein language model embedding). These vectors are stacked to form a sequence used as input for the deep learning model. (B) Schematic of the PathogenFinder2 neural network, comprising two main components: an adaptation of the ConvNext (Liu *et al.* 2022) image classifier for sequential data (consisting of 1D Convolutions) and a Bahdanau attention (Bahdanau *et al.* 2016) layer to account for unequal protein importance. These components, free from Transformer model length limitations, enable the reporting of pathogenic capacity, protein importance, and genome pathogenic-related embeddings.

PathogenFinder2 performs whole-genome phenotype prediction through a four-step process (Fig. 2A) which transforms a bacterial genome into a suitable input for a neural network. Initially, Prodigal (Hyatt *et al*. 2010) predicts a collection of proteins from the genome, each of which is embedded using the ProtT5 (Elnaggar *et al*. 2022) protein language model. This language model was chosen for its performance in other tasks and the size of its embeddings. To prevent out-of-memory errors from long protein sequences, the maximum length of a protein was set to 9.920 amino acids. Longer sequences are split into overlapping segments before embedding, ensuring PathogenFinder2 fits within a 24GB GPU for inference. The maximum length can be adjusted for different infrastructures, though this may affect the model’s accuracy. Embeddings are summed across the amino acid dimension, yielding a 1.024-dimensional vector per protein. These vectors are stacked to form a 2D input matrix of shape *Nx1024*, where *N* is the number of predicted proteins. As Prodigal predicts the proteins from the genome in the same order they appear in the FASTA file, the proteins on the input matrix retain the same arrangement as they appear in the genome. Because of that, the same protein sorting exists on the input of the neural network as in the bacterial genome, although ignoring the distance between genes. While our experience suggests that preserving this order facilitates better model fitting, its impact is outside the scope of this study.

The neural network (Fig. 2B) comprises three main components: convolutional learning, attention layer and sigmoid output layer. The first, inspired by vision models, is a modified version of the state-of-the-art model ConvNext (Liu *et al*. 2022), to address the variable and relatively large length of the input (e.g., *Escherichia coli* has approximately 4.288 coding genes). Moreover, to address local relationships (e.g., operons or mobile elements) by kernel learning. As the modified ConvNext blocks maintain the N dimension of the input, the second component, an additive or Bahdanau attention layer (Bahdanau *et al*. 2016), assigns importance weights to each protein and collapses that dimension. To provide structured information to the neural network, the number of predicted proteins is embedded using a positional encoding-like architecture concatenated with the attention layer output. Integration of this information was optimized via hyperparameter tuning (Supplementary Material 1, Table 1, available as supplementary data at *Bioinformatics* online). This concatenated output is fed into a sigmoid layer for classification and serves as the bacterial genome embedding. A more detailed description of the neural network is in Supplementary Material 1, Fig. 2, available as supplementary data at *Bioinformatics* online.

PathogenFinder2's neural network model is an ensemble of four networks, each trained on different training/validation splits. The final prediction is the averaged output of the four networks.2.2.1 PathogenFinder2 attention score and protein highlight.

PathogenFinder2 outputs four attention scores per protein, one from each neural network attention module. These scores are interpreted as a ranking of protein relevance for the pathogenic capacity prediction. To contextualize the relevance of the protein sequences, PathogenFinder2 optionally maps the bacterial proteome to either UniRef50 (Suzek *et al*. 2007) or the reviewed sequences of UniProtKB/Swiss-Prot  (Bateman *et al.* 2025). Mapping is performed with DIAMOND (Buchfink *et al*. 2015) using default parameters and the *faster* mode, returning the top-scoring hit for each query protein.

UniRef50 offers broader coverage, but provides limited metadata and is computationally expensive. Therefore, PathogenFinder2 aligns only the top 20 protein sequences ranked by each of the four attention scores.

In contrast, Swiss-Prot provides richer annotations and faster alignment; however, its smaller size may reduce proteome coverage. Because Swiss-Prot has more standardized metadata, which includes GO terms, Gene Set Enrichment Analysis (GSEA) (Subramanian *et al*. 2005) is used to identify protein families, functions, and pathways strongly highlighted by the attention mechanism. Proteins are ranked by the maximum attention score across the four attention modules, and a pre-ranked GSEA is performed. Protein sets are defined by the Swiss-Prot identifier prefix (letters before underscore), or the associated GO term (Biological Processes) (The Gene Ontology Consortium 2021). GSEA is implemented with GSEApy Python package (Fang *et al*. 2023), using default settings, with the number of permutations increased to 10.000.

These two procedures (on a UniRef50 dataset downloaded on 07/01/2025 and UniProt downloaded on 08/11/2025) were applied to Test-NovelSpecies, Test-2024Strains and the metagenome-assembled genomes (MAGs) from globally collected sewage samples (Jespersen *et al*. 2023).

#### 2.2.2 PathogenFinder2 hyperparameter optimization and training

Initial model parameters (e.g., weight initialization, kernel size, batch size, optimizer) were selected based on prior studies. Seven additional parameters were optimized with the Tree-structured Parzen Estimator (TPE) (Bergstra *et al*. 2011) algorithm on Optuna (Akiba *et al.* 2019), aiming to maximize the Matthews Correlation Coefficient (MCC) (Matthews 1975) of the neural network model ensemble. This was done with a 4-fold cross-validation, where each model was trained for 60 epochs and the best MCC value on the validation set was used to calculate the ensemble MCC. Only the final 50 epochs were considered to avoid early training instability. The TPE algorithm guided 50 trials, followed by 10 manually informed ones, yielding only minor improvements over initial settings.

Bucketing (Khomenko *et al*. 2016) and batch stratification were applied throughout. Training used the RAdam (Liu *et al*. 2021) optimizer with a warm-up period and a scheduler that reduced the learning rate when the loss increased. The final models were trained for 200 epochs, selecting the weights from the best validation epoch.

Models were implemented in PyTorch (Paszke *et al*. 2019) and trained on GPUs Tesla NVidia A100 with 40 GB HBMI2, with CPUs AMD Epyc 7H12.

### 2.3 Other models

#### 2.3.1 Previous pathogen predictors

We tested the previous protein-based bacterial pathogen predictors PathogenFinder1, BacPacs and WSPC, as well as the read-based predictor DeePac on the Test-NovelSpecies and Test-2024Strains.

Prodigal was run with standard setup to produce the protein predictions for PathogenFinder1 and BacPacs methods. For PathogenFinder1, the same code that runs behind the WebServer (https://cge.food.dtu.dk/services/PathogenFinder/) was used (provided by the department that hosts it), with the standard setup and with the “All” database (database without any phylum specified). The probability value given by the algorithm was used to build the ROC and calibration curves.

For BacPacs, we followed the instructions of the section “Predicting data using bacpacs pre-trained model” available from their repository (https://github.com/barashe/bacpacs). As BacPacs only provides a label as output, we used the probability estimation for OvR logistic regression (*_predict_proba_lr*) of the linearSVC that the model uses for predicting pathogenic capacity. The probabilities are computed as: $\frac{1}{1+e^{-\mathrm{decisionfunction}(x)}}$

For WSPC, we followed the instructions available on their repository (https://github.com/shakedna1/wspc_rep) using their existing model. We obtained the PATRIC Global Protein Families (PGFams), required by the model, through BV-BRC’s Genome Annotation Service (https://www.bv-brc.org/app/Annotation) as described in the repository. As we have not given any information to the other models about the taxonomy of the test set, we set the Taxonomy ID to 2 (bacteria).

For DeePac, we followed the instructions described in their repository (https://gitlab.com/rki_bioinformatics/DeePaC). As it requires the input to be reads, we used their method to produce pseudoreads (*deepac gwpa fragment*), before predicting bacterial pathogenic capacity.

BacPacs, WSPC and PathogenFinder1 models were tested on a Linux system on Intel(R) Xeon(R) CPU E7-8870 v4 @ 2.10 GHz. DeePac was tested on a NVIDIA Tesla V100 SXM2 with 32 GB GPUs with PCIE.

#### 2.3.2 Baseline pathogen predictors

The baseline method *Kmer-Patho* is an adaptation of the KmerFinder taxonomy detection method, which examines the number of co-occurring *k*-mers (Hasman *et al*. 2014). We used the code available in the repository (https://bitbucket.org/genomicepidemiology/kmerfinder.git). To shape the PathogenFinder2 dataset so it can be used by our tailored KmerFinder, we indexed the development subset (without overlap with Test-NovelSpecies) using KMA with “*kma index -i <in.fna> -o <OUT> -Sparse ATG*,” as used in the database behind the webserver https://cge.food.dtu.dk/services/KmerFinder/. Unlike the species predictor, each sequence was only defined by its pathogenic capacity. The prediction was then done taking the phenotype of the closest sequence. To provide a continuous output (in order to produce the ROC and Calibration curves), the query coverage of the hit on the database was used.

The other baseline method created was a version of PathogenFinder1 with the new database. We followed the procedure described by the authors and that is implemented on the code behind the webserver https://cge.food.dtu.dk/services/PathogenFinder. Due to the significant increase in the protein database size, CD-Hit (Li and Godzik 2006) was replaced by the state-of-the-art clustering method MMSeqs2 (Steinegger and Söding 2017) for building the database (clustering proteins, using the *linclust* clustering strategy), as well as for searching for matches (using the *search* module).

Both baseline models were created and tested on a Linux system with an Intel Xeon E7-8870 v4 @ 2.10 GHz CPU.

## 3 Results

### 3.1 PathogenFinder2 excels in predicting novel pathogens

**Figure 3:**
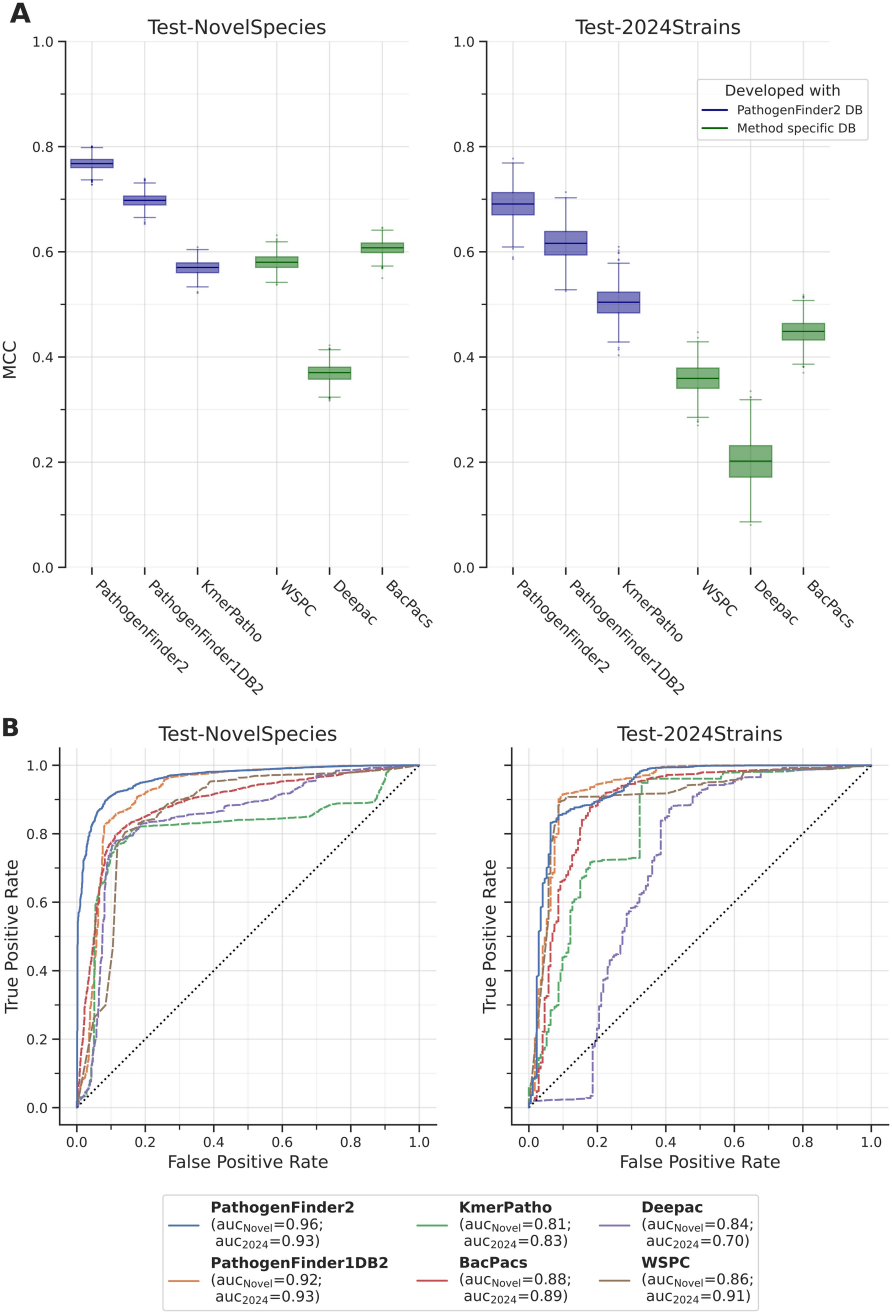
Performance measures of PathogenFinder2, Baseline Methods, and Previous State-of-the-Art Methods. (A) Box plots showing the 5 quartiles of the MCC values from bootstrapping on the different test sets. (B) ROC curves with AUC in the legend. On the left panels, Test-NovelSpecies (3.310 pathogenic, 976 non-pathogenic), with bacterial species not included in training PathogenFinder2 or baseline models. On the right panels, Test-2024Strains (3.704 pathogenic, 176 non-pathogenic) with bacteria annotated a year after PathogenFinder2 dataset creation. Besides PathogenFinder2, we evaluated two baseline models trained with the PathogenFinder2 database, PathogenFinder1DB2 (PathogenFinder1 trained with the new dataset) and KmerPatho (a method based on the k-mer based species predictor KmerFinder (Hasman *et al.* 2014)), and four previous state-of-the-art pathogen predictors. The methods were executed usingtheir pretrained configuration or database, as specified in their repositories. Further details are provided in the Methods section. The original PathogenFinder1 failed to generate any result for 1.660 genomes in Test-NovelSpecies and 2.189 in Test-2024Strains, so it was excluded from the comparison.

Our novel model, PathogenFinder2, achieved a higher Matthews correlation coefficient (MCC) compared to both the baseline models trained with the new dataset (PathogenFinder1DB2, KmerPatho) and previous state-of-the-art models (PathogenFinder1 (Cosentino *et al*. 2013), WSPC (Naor-Hoffmann *et al*. 2022), BacPacs (Barash *et al*. 2019), DeePac (Bartoszewicz *et al*. 2020)) on new species (Test-NovelSpecies). For the dataset allowing for leakage (similarity between training and testing allowed, Test-2024Strains), the MCC for PathogenFinder2 remained higher than for PathogenFinder1DB2; however, this difference was not statistically significant according to the 95% confidence interval (CI, i.e., within ±1.96 standard errors; Fig. 3A). PathogenFinder2 also demonstrated a superior ROC curve and higher AUC compared to other methods in Test-NovelSpecies (Fig. 3B). On the test set permitting similarity, PathogenFinder2 exhibited metrics comparable to PathogenFinder1DB2, though it had a higher true positive rate when the false positive rate was near zero. The species-based baseline method, KmerPatho, performed significantly worse than both PathogenFinder2 and PathogenFinder1DB2 in terms of MCC and AUC, especially when there was no species overlap between its database and the test set, in which case it was also outperformed by previous pathogenic predictor methods.

In terms of sensitivity, PathogenFinder2 was only surpassed by DeePac on Test-NovelSpecies (Supplementary Material 1, Fig. 3, available as supplementary data at *Bioinformatics* online). However, DeePac exhibited very low specificity (Supplementary Material 1, Fig. 3, available as supplementary data at *Bioinformatics* online) due to a high rate of false positives (Supplementary Material 1, Fig. 4, available as supplementary data at *Bioinformatics* online). BacPacs was the only method with higher specificity than PathogenFinder2, although this difference was again not statistically significant. Additionally, PathogenFinder2 showed a remarkably well-calibrated output for a deep learning model, particularly in the more extreme output regions (0–0.3 and 0.8–1 in Supplementary Material 1, Fig. 5, available as supplementary data at *Bioinformatics* online).

**Figure 4:**
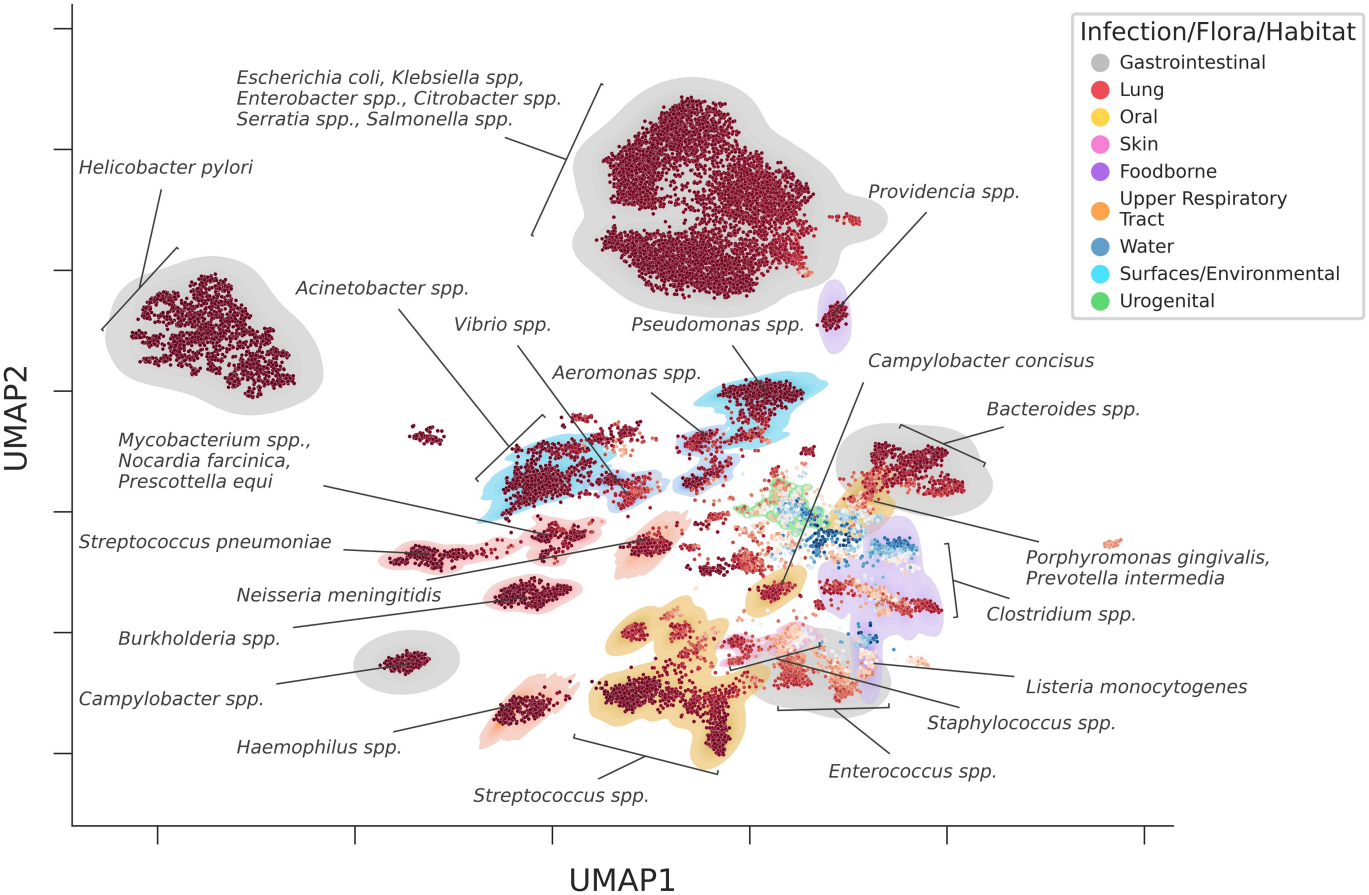
Projection of the embeddings of all bacterial sequences in the PathogenFinder2 database with pathogenic capacity, illustrating the Bacterial Pathogenic Capacity Landscape. Each dot represents a bacterial sequence that was embedded using PathogenFinder2 and plotted in a UMAP (McInnes *et al.* 2020) of two dimensions. The dots are colored depending on the PathogenFinder2 pathogenic capacity prediction, with red being positive and blue negative The distribution representing the infection, flora or habitat of the bacteria was drawn with a kernel density estimation, and colored through a process of clustering with HDBScan (Campello *et al.* 2013) (as shown in the Figure S6 in Supplementary Material 1) and manual annotation based on bibliography. Some species and genera of interest are indicated with arrows.

### 3.2 Attention highlights proteins related to pathogenic capacity

Mapping to the UniRef50 dataset showed a tendency for which most proteins from the bacteria predicted with pathogenic capacity in Test-NovelSpecies and Test-2024Strains sets (Supplementary Material 2, available as supplementary data at *Bioinformatics* online) fell into various virulence factor categories described in previous literature (Liu *et al*. 2022) (Supplementary Material 1, Table 2, available as supplementary data at *Bioinformatics* online), as well as several uncharacterized proteins. In the bacteria predicted without pathogenic capacity from our Test-NovelSpecies and Test-2024Strains, most proteins lacked an annotated function. But among the proteins annotated with a function, we found antimicrobial producing genes, protein antidotes, glucosidases for degrading lactose and cellulose, and proteins involved in the shikimate pathway and chlorophyll production.

**Table 2:** Protein identifiers and functions highlighted by attention scores using preranked GSEA on bacteria predicted with pathogenic capacity.

Protein Identifier	GO term
*Adhesion & epithelial interaction:* attaching and effacing proteins, BmaC autotransporter, pilin, fimbrial Usher proteins. *Surface structures & host-matrix interaction:* Muramidase-released protein, S-layer protein, major surface antigen 4, extracellular matrix-binding protein Ebh. *Barrier degradation:* muramidase-like enzyme (lysozyme), sialidase A. *Biofilm & exopolysaccharide synthesis:* dextransucrase, glucosyltransferase-I & -V, antigen 43 autotransporter adhesin, accumulation-associated protein. *Secretion & toxins:* tRNAase CdiA, Rhs, type VI secretion system. *Nutrient transport and permeability:* TonB-dependent receptors (P3, SusC, P26), porin B. *Iron acquisition:* hemoglobin/hemoglobin-haptoglobin-binding protein. *Regulatory:* Cyclic di-GMP phosphodiesterase. *Mobility & chemotaxis:* chemotaxis related protein, flagellin. *Anaerobic lifestyle:* Oxaloacetate decarboxylase alpha chain. *DNA mobility & DNA maintenance:* Transposases, insertion sequence elements, tyrosine recombinase, crossover junction endodeoxyribonuclease, recombination-promoting nuclease, conjugation protein TraI, TraC protein, exodeoxyribonuclease 8. *Cell envelope:* Endo-alpha-N-acetylgalactosaminidase, Mannose-1-phosphate guanylyltransferase, ATP-dependent protease, ATP-dependent zinc metalloprotease. *Other:* 2,3-bisphosphoglycerate-dependent phosphoglycerate mutase bacteria, thioredoxin, Inner membrane protein YeeR, Iron-regulated protein FrpA, E3 ubiquitin-protein ligase, Methionyl-tRNA formyltransferase. *Viral proteins:* tail fiber, protein LomR, antitermination protein Q	Peptide transport* (Uncharacterized lipoprotein) ''de novo’ IMP biosynthetic process (FGAM synthase) Glycolipid biosynthetic process (Probable Ac1PIM4-binding protein) Protein secretion by the type VI secretion system (T6SS spike protein) L-phenylalanine catabolic process* (Primary amine oxidase) 3,4-dihydroxybenzoate biosynthetic process (Quinate/shikimate dehydrogenase) Response to stress (Universal stress protein Rv2028c) Anaerobic respiration (DMSO reductase) DNA integration (ATP-dependent protease) Menaquinone biosynthetic process (SEPHCHC synthase) Iron-sulfur cluster assembly (Iron-sulfur cluster assembly protein) Bacterial-type flagellum assembly* (Flagellar hook-associated protein 2) Positive regulation of DNA-templated transcription (Uncharacterized protein Rv2242) Sporulation resulting in formation of a cellular spore (Pesticidal crystal protein Cry22Aa). L-histidine biosynthetic process (IGP synthase cyclase subunit) Iron ion transport* (Hemin transport protein) Catabolic process (OHED hydratase) Lipid catabolic process* (Lipase chaperone) Chitin catabolic process (Chitobiase) Heme import across plasma membrane (Heme exporter protein D) Plasmid partitioning (Protein SopA) Cell adhesion (Intimin) CRISPR-cas system (CRISPR-associated endoribonuclease Cas2) Viral release from host cell by cytolysis (Prophage Rz endopeptidase) rRNA base methylation (16S rRNA methyltransferase F) Amino acid activation for nonribosomal peptide biosynthetic process (PTT synthase PhsB) Translational elongation (EF-Tu 1) Response to hydrogen peroxide (Hydroxylamine reductase) Secondary metabolite biosynthetic process (AMB antimetabolite synthetase) Cellular response to oxygen levels (Uncharacterized protein YbdM) Lipid biosynthetic process (Tyrocidine synthase 1

Protein Identifiers and GO terms showing significant positive enrichment (FDR *q*-value < 0.01) based on attention scores in bacteria predicted with pathogenic capacity in the Test-NovelSpecies and Test-2024Strains datasets are listed. Protein Identifiers are grouped by functional categories, including classes commonly annotated as virulence-associated. The order of appearance does not reflect enrichment magnitude. Uncharacterized proteins were excluded. GO terms appear sorted by their median Normalized Enrichment Score (NES). When a term appears in both test sets (marked with an asterisk), only the instance with the higher median NES is shown. Parentheses indicate the leading edge protein (gene that contributed the most to the enrichment signal) for each GO term. It is noticeable some inconsistencies on the GO annotations from the Swiss-Prot database, such as “Pesticidal crystal protein Cry22Aa” being annotated as “Sporulation resulting in formation of a cellular spore,” being a toxin aimed at insects.

Mapping to the Swiss-Prot dataset followed by GSEA on protein identifiers and GO terms revealed broadly concordant enrichment patterns. In bacteria predicted to have pathogenic capacity, significantly enriched protein identifiers included proteins annotated as virulence-associated, such as toxins, proteins involved in adhesion and host-epithelium interactions, as well as proteins associated with biofilm formation and barrier degradation (Table 2). Enriched GO terms reflected similar categories.

Some enriched identifiers have a more indirect relationship to pathogenic capacity (such as iron intake), or even correspond to core metabolic functions (e.g. nutrient uptake and cell envelope biogenesis). Genes related to genetic mobility and proteins of prophage origin are also significantly enriched.

Most of the significantly positively enriched protein identifiers and GO terms from bacteria predicted without pathogenic capacity (Supplementary Material, Table 3, available as supplementary data at *Bioinformatics* online) correspond to core metabolic proteins commonly observed in environmental and plant-associated bacteria. However, some of those core metabolic proteins also appear highlighted in bacteria predicted with pathogenic capacity (for example, TonB receptors). Moreover, a few identifiers that are commonly associated with pathogenic capacity (for example, internalins) were significantly enriched in bacteria predicted without pathogenic capacity (Supplementary Material, Table 3, available as supplementary data at *Bioinformatics* online).

**Table 3:** Pathogenic clusters in metagenomic samples.

Cluster	Taxonomy Predicted	Proteins Highlighted	Protein Identifiers Highlighted	GO Terms Highlighted
0 (6 samples)	Brachymonas sp.	Long-chain fatty acid transporter, hemagglutinin, toxins, adhesion/adhesins, porin	–	Establishment of protein localization, DNA recombination, intracellular chemical homeostasis, membrane organization; protein transport, monoatomic ion transport.
9 (55 samples)	Pseudomonas sp.	Toxins, RHS domain, peroxidases, flagellin, fimbria, quinate dehydrogenase, chemotaxis	Ferripyoverdine receptor, Porin, tRNA nuclease CdiA, Pyruvate synthase subunit PorD	Type IV pilus assembly, basic amino acid transport, Negative regulation of DNA-templated transcription; protein maturation, biological process involved in interspecies interaction between organisms
10 (26 samples)	Escherichia coli, Erwinia rhapontici, Serratia fonticola, Rahnella spp., Enterobacter cloacae, Citrobacter freundii, …	HemS, anaerobic metabolism, intimin, KWG domain, fimbrial, flagellin, alpha-amylase	–	Iron ion transport, anaerobic respiration, 2'-deoxyribonucleotide biosynthetic process, iron-sulfur cluster assembly, ferric-hydroxamate import into cell, pilus assembly; cell projection organization, secondary metabolite biosynthetic process.
14 (82 samples)	*Acinetobacter sp*., *Mesocricetibacter sp.*, *Chelonobacter sp*.	Glucose metabolism (PQQ dependent), T6SS.	Toxin CdiA, Lysozyme B, Cold shock protein, Ribose import permease protein	Type IV pilus assembly, Protein transport, Protein transport, L-tryptophan biosynthetic proces; cell projection organization, transposition, biological process involved in interspecies interaction between organisms.
5 (39 samples)	Streptococcus sp.	Pullulanase, LPXTG domains, nucleases, general stress protein, penicillin-binding protein	Ribosome- binding protein, Uncharacterized protein y4pE	De novo’ IMP biosynthetic process, peptidoglycan biosynthetic process, peptide transport, DNA recombination; response to stimulus, protein folding.
40 (64 samples)	Ottowia sp., Thermomonas sp., Thauera sp., Castellaniella sp., Sphaerotilus sp. …	Pili, anchor protein, flagellin, hemagglutinin, copper resistance, chemotaxis	Chemotaxis, Diguanylate cyclase DgcP, Bifunctional diguanylate cyclase/cyclic di-GMP phosphodiesterase MucR	Butyrate metabolic process, type IV pilus-dependent motility, iron ion transport, heme biosynthetic process; cell motility, defense response to other organism, protein-containing complex assembly
29 (22 samples)	Brachymonas chironomi, Brachymonas denitrificans	Pilus, VCBS, NlpC/P60 protein		Protein transport, DNA repair, nucleotide-excision repair, Gram-negative-bacterium-type cell outer membrane assembly; nitrogen cycle metabolic process, xenobiotic catabolic process

In this table we find the clusters with predicted pathogenic capacity bacteria found in metagenomic samples (Supplementary Material 1, Figure 7, available as supplementary data at *Bioinformatics* online), showing their most common predicted taxonomy, as well as the proteins highlighted by the attention vector. Mapping these clusters onto the phylogenetic tree from (Jespersen *et al.* 2023) revealed that pathogenic phenotypes were not consistently homogeneous within each clade (Supplementary Material 1, Figure 8, available as supplementary data at *Bioinformatics* online). The column Proteins Highlighted shows top matches against UniRef50. Columns Protein Identifiers Highlighted and GO Terms Highlighted include identifier groups and GO terms with the lowest FDR q-values contributing positively to pathogenicity. Except for toxin CDA in cluster 14, none met the FDR threshold (0,01) for statistical relevance.

### 3.3 The bacterial pathogenic capacity landscape

We also offer an initial overview of the human *Bacterial Pathogenic Capacity Landscape* with the bacteria with pathogenic capacity of our dataset (Fig. 4). We use PathogenFinder2’s capacity of embedding a genome into a fixed-length vector based on its pathogenic capacity. As expected, there appears to be correlation between closeness and the phylogenetic proximity of the species. However, we also observe patterns indicating that the embeddings contain additional information, such as the site of infection. For example, *Campylobacter concisus* is distinctly separated from other *Campylobacter* species that infect the gastrointestinal tract, but closer to skin flora. Similarly, *Streptococcus pneumoniae* is grouped with bacteria that cause lung infections rather than with other *Streptococcus* species (including *S. pyogenes* and *S. agalactiae*), as part of the oral flora.

Common oral pathogens such as *Porphyromonas gingivalis*, *Prevotella intermedia*, and *C. concisus* appear closely related to foodborne species from the *Clostridium* genus, like *C. perfringens*, *C. difficile*, and *C. botulinum*. These oral pathogens surround a miscellaneous group of bacteria known for degrading and fermenting biological material (such as the *Bacteroides* genus, part of the gut microbiota but isolated from infections in soft tissues and bacteremia), suggesting metabolic similarities between oral and foodborne pathogens rather than with oral flora. The latter, mainly part of the *Streptococcus* genus, have been reported to cause infections in other body parts, such as bacteremia or endocarditis. They are located next to species colonizing the upper respiratory tract from the *Haemophilus* genus (like *H. influenzae* or *H. haemolyticus*), which also cluster together with *Pasteurella multocida*. Although *P. multocida* is not part of the human upper respiratory tract flora, it is of certain animals and can be transmitted through their bites (Arons *et al*. 1982). Another upper respiratory tract flora member, *Neisseria meningitidis*, appears close to lung infection pathogens. Although rare, *N. meningitidis* can cause pneumonia (Feldman and Anderson 2019), which may explain this distribution.

The embedded representation of the bacterial proteome does not always correlate with the site of infection, but rather their habitat. For instance, *Vibrio* species like *V. parahaemolyticus* and *V. cholerae* appear closely to common nosocomial pathogens such as *Acinetobacter spp*. Or the known foodborne pathogen *Listeria monocytogenes* is placed alongside *Enterococcus casseliflavu*s, a species that has only been seen to cause diseases in immunocompromised patients (Yoshino 2023). However, the capacity of some strains of this species to prevent *L. monocytogenes* infection by competing with their colonization using bacteriocins (Sabia *et al*. 2003), could explain their proximity in the landscape. Moreover, *Klebsiella pneumoniae*, part of the gut microbiota but able to infect pulmonary tissues, appears in a cluster alongside gastrointestinal pathogens. The dimensions and spread of this cluster could suggest genomic similarity, due to, for example, the exchange of mobile elements (Kent *et al*. 2020). Other clusters of bacteria that cause gastrointestinal diseases, like *Helicobacter pylori* or *Campylobacter spp.*, show very different conformations. While the cluster of the latter, with two species (*C. jejuni* and *C. coli*), forms a dense cluster, the one with only one species, *H. pylori*, appears dispersed, likely due to the notable genomic variability of the species (Ailloud *et al*. 2021).

### 3.4 Practical case: bacterial pathogenic capacity prediction on metagenomic samples

PathogenFinder2 was employed to analyze 2.739 metagenomic assembled genomes (MAGs), many of which were completely novel taxa, from a previous study on sewage bacterial communities (Jespersen *et al*. 2023). The analysis predicted that 1,839 bacteria lacked pathogenic capacity, while 370 were potentially capable of infecting humans under certain conditions. The remaining 530 MAGs were classified as Uncertain due to the neural network predictions being close to the 0.5 threshold.

Using the embeddings of the genomes, we identified seven groups of potential pathogens (Supplementary Material 1, Figs. 7 and 8, available as supplementary data at *Bioinformatics* online), and their closest known taxa (Table 3), including some not previously reported as human pathogenic (such as *Brachymonas*). Examination of proteins highlighted by attention scores reveals known virulence factors (such as toxins, adhesins, and proteins involved in intracellular invasion). However, preranked GSEA did not identify any significantly enriched pathogenic capacity-related group or functions due to high FDR q-values.

## 4 Discussion

PathogenFinder2 does not aim to predict bacterial pathogenicity, as the interaction outcome between bacteria and humans also depends on the latter (Casadevall and Pirofski 2002, Méthot and Alizon 2014). Instead, we redefined the objective to predict bacterial pathogenic capacity, which is solely determined by the bacteria. The dataset used to train the model was designed to reflect this definition, as it would shape the deep learning model accordingly. Genomes were classified as “pathogenic” if they had caused any human infections, regardless of virulence, host conditions, or incidence. This broader definition should be considered when using PathogenFinder2, as it may predict bacteria with pathogenic capacity that might require specific host conditions to cause harm. For instance, there is no difference in the predictions of PathogenFinder2 (and other pathogen predictors used in this study, Supplementary Material 1, Fig. 9, available as supplementary data at *Bioinformatics* online) on *E. coli* strains found in diarrheal and non-diarrheal patients (Hazen *et al*. 2023). Naturally, the pathogenic capacity predictors might not be fine-grained enough to detect minimal genetic differences between pathogenic and nonpathogenic *E. coli* (Koli *et al*. 2011). But, being all predicted with pathogenic capacity, the difference on the interaction outcome might not be due to different pathogenic capacity, but for reasons only reliant on the host.

Additionally, labeling bacteria as pathogenic based on their isolation from an infection site carries the risk of mislabeling non-pathogenic microbiota, as they might be present in the affected area but not causing harm. In fact, the presence of noise in bacterial database annotations is unavoidable. Previous attempts to create pathogen predictors have tried to minimize this issue by arbitrarily selecting one phenotype for some heterogeneous species (Bartoszewicz *et al*. 2020, Naor-Hoffmann *et al*. 2022). However, this approach does not acknowledge the existence of species with heterogeneous pathogenic capacity among their strains. To avoid simplifying the neural network’s objective to a mere species predictor, we relied on genomic annotations provided by researchers uploading genomes to publicly available databases, allowing similar genomes with different phenotypes. Although this might introduce some noise into the PathogenFinder2 dataset, we believe the model has overcome these noisy labels. For example, the sequences predominantly predicted as non-pathogenic shown in the *Bacterial Pathogenic Capacity Landscape* (Fig. 4) by PathogenFinder2 mainly consist of bacteria, such as *Bacteroides* or *Lactobacillus*, found in the human microbiome that populates common infection sites of other bacteria.

Another consideration when working with bacterial pathogenic capacity is the potential bias in our dataset’s subset of bacteria without pathogenic capacity. Besides extremophiles unable to live in the human body, we required that these bacteria were frequently in contact with humans without reported infections. This might have skewed the subset and may not represent reality. This compromise is unavoidable, as creating new data would require testing bacterial pathogenic capacity in humans. Additionally, the ratio of bacteria with and without pathogenic capacity in the database may not reflect real ecosystems. For instance, only 13% of bacterial genomes isolated from sewage (Jespersen *et al*. 2023) were predicted to have pathogenic capacity, compared to 76,9% in our training dataset. Although we cannot generalize the ratio found in sewage to other ecosystems, it demonstrates our model’s ability to learn beyond the dataset imbalance.

Our model outperforms the previous state-of-the-art pathogenic capacity predictors on novel species, with balance between specificity and sensitivity in its predictions. This is particularly noteworthy given that PathogenFinder2 was trained without exposure to these species, unlike the previous models that may have overlapping species between their training sets and our test sets. PathogenFinder2 effectively overcomes data-related challenges that our baseline models struggle with, showing that our model’s success can not only be attributed to the creation of the largest bacterial pathogen dataset created, but also to the model’s architecture. For instance, PathogenFinder1DB2 (PathogenFinder1 trained with the new dataset), like any alignment-based model, suffers from lower accuracy on unseen species due to dataset incompleteness, in particular when predicting the under-represented label (non-pathogenic). Additionally, KmerPatho underperforms compared not only to PathogenFinder2, but also against older models, particularly on unseen species, indicating that pathogenic capacity prediction cannot be achieved using species prediction models.

PathogenFinder2 combines the feature of not requiring a tailored database of proteins related to pathogenic capacity (as in previous read-based models), while still identifying proteins of interest for its predictions (as in previous protein-based models). Although attention scores as explanation signals for pathogenic capacity pose challenges (Jain and Wallace 2019, Wiegreffe and Pinter 2019), they might offer valuable insights for surveillance programs and researchers on where, and hence which proteins, the model has focused to make the prediction (Table 2, and Supplementary Material 1, Table 3, available as supplementary data at *Bioinformatics* online). The lack of strict causality in attention weights is not necessarily a drawback, as it might provide insight into candidate virulence factors and metabolic infectious pathways not found by more traditional methods.

Proteins contributing to predictions can be ranked by attention score following alignment to UniRef50 (Supplementary Material 2, available as supplementary data at *Bioinformatics* online), or mapped to Swiss-Prot, allowing for the use of preranked GSEA for statistical significance analysis. In bacteria predicted as pathogenic in our test sets (Table 2), enriched proteins and pathways include canonical virulence-associated processes as well as indirectly related traits, such as iron acquisition (Cassat and Skaar 2013) and glucose intake (Passalacqua *et al*. 2016). Enzymes involved in DNA integration, mobility and genetic stability appear repeatedly, suggesting the importance of genomic variability and mutation rates for bacterial adaptation to hosts, as previously found for *Salmonella enterica* and *E. coli* (Pan *et al*. 2022, Swings *et al*. 2017).

However, some protein families and biological processes expected (based on prior literature) to be related to a certain pathogenic phenotype appear highlighted in bacteria predicted with the opposite capacity. For example, pathways related to plant-related bacteria in bacteria predicted with pathogenic capacity (Table 2), as well as proteins directly involved in the invasion of host cells (internalins) in bacteria predicted without pathogenic capacity (Supplementary Material 1, Table 3, available as supplementary data at *Bioinformatics* online). This reflects a key interpretative constraint: high attention scores identify proteins that are informative for discrimination between pathogenic and non-pathogenic capacity, with samples containing high scores in support of both phenotypes. For example, internalins were present in *L. monocytogenes* strains misclassified as without pathogenic capacity with borderline scores, indicating that attention should be interpreted as highlighting influential proteins, rather than implying a direct association with the predicted pathogenic capacity.

Moreover, while GSEA adds statistical support to the attention scores, it is limited by the sometimes scarce and uneven coverage in the Swiss-Prot dataset (Supplementary Material 1, Fig. 10, available as supplementary data at *Bioinformatics* online), likely explaining the lack of relevant enriched protein sets on the clusters of MAGs, due to containing less-studied taxa. Additionally, grouping proteins by identifiers or GO terms may exclude single pathogenicity-relevant proteins and obscure associations through overly broad or erroneous annotations.

Given the limitations of attention methods on feature importance attribution explained above, and the complexity of the pathogenic capacity phenotype, the significance of the proteins highlighted requires further investigation, as the insights provided on the mechanisms for host-damage might be limited. However, this approach can be used as an exploratory tool, as identifying candidate proteins and metabolic pathways can provide valuable hypotheses for infectious disease research. In fact, we believe that this approach could complement other traditional approaches, such as comparative genomics and genetic screening, by directing research toward genes related to pathogenic capacity before their precise roles are elucidated (Hu *et al*. 2011, Rappuoli *et al*. 2025). Where conserved pathways are implicated, this may include the development of broad-spectrum antimicrobial strategies.

PathogenFinder2 can also be used to create an embedding of a bacterium related to its pathogenic capacity. This has allowed us to produce the initial mapping of the *Bacterial Pathogenic Capacity Landscape*, providing insights into the diverse mechanisms by which bacteria inflict harm on human hosts, particularly in the common site of infection and habitat, which can be of great impact when phenotyping a bacteria. Additionally, it can aid in the study of rare and under-researched pathogens, as well as interactions between bacterial species. Surprisingly, all those features encapsulated in PathogenFinder2's embeddings were not part of the training data. This opens the door to future iterations of our model incorporating this information when predicting pathogenic capacity, offering a more realistic and informative output regarding the threat certain bacteria pose to humans.

The aim of PathogenFinder2 is to provide a tool for surveillance of novel pathogens and the study of human infections. With the resourceful output of our model, available for local use or through our portal (http://genepi.dk/pathogenfinder2), we believe it will enable more targeted early detection of future pathogens, as well as the development of treatment strategies and vaccinations even before infections are observed, as demonstrated on the analysis of the MAGs from sewage (Jespersen *et al*. 2023). Moreover, to the best of our knowledge, PathogenFinder2 represents the first successful attempt to utilize pLMs for the prediction of whole genomes as input. These analyses can now be expanded to other kingdoms, as well as other phenotypes that affect the majority of the genome with respect to bacterial pathogenic capacity.

## Supplementary Material

btag129_Supplementary_Data

## Data Availability

The data underlying this article are available at the PathogenFinder2 repository, in https://github.com/genomicepidemiology/PathogenFinder2, as well as a FigShare repositories: https://doi.org/10.6084/m9.figshare.28779440.v1 (data), and https://doi.org/10.6084/m9.figshare.29413277.v3 (code).
