# Supplementary material for: Whole-genome prediction of bacterial pathogenic capacity on novel bacteria using protein language models with PathogenFinder2
Source: Bioinformatics. 2026 May 28;42(5):btag129. doi: 10.1093/bioinformatics/btag129 (PMC13218381; doi:10.1093/bioinformatics/btag129)
Supplement: btag129_Supplementary_Data [file btag129_supplementary_data.zip › SupplemenaryMaterial1-- Whole-genome prediction of bacterial pathogenic capacity on novel bacteria using protein language models, with PathogenFinder2 .pdf]

Supplementary Material 1

Whole-genome prediction of bacterial pathogenic capacity on novel bacteria using protein language models, with PathogenFinder2

Alfred Ferrer Florensa[1,*], Jose Juan Almagro Armenteros[2], Rolf Sommer Kaas[1], Philip Thomas Lanken Conradsen Clausen[1], Henrik Nielsen[3], Burkhard Rost[4] and Frank Møller Aarestrup[1]

[1] Research Group for Genomic Epidemiology, National Food Institute, Technical University of Denmark, Kongens Lyngby, 2800, Denmark

[2] Informatics and Predictive Sciences Research, Bristol Myers Squibb Company, Sevilla, 41092, Spain

[3] Bioinformatics, Department of Health Technology, Technical University of Denmark, Kongens Lyngby, 2800, Denmark

[4] Rostlab, Department of Bioinformatics and Computational Biology, Technical University of Munich, Munich, 85748, Germany

*To whom correspondence should be addressed.

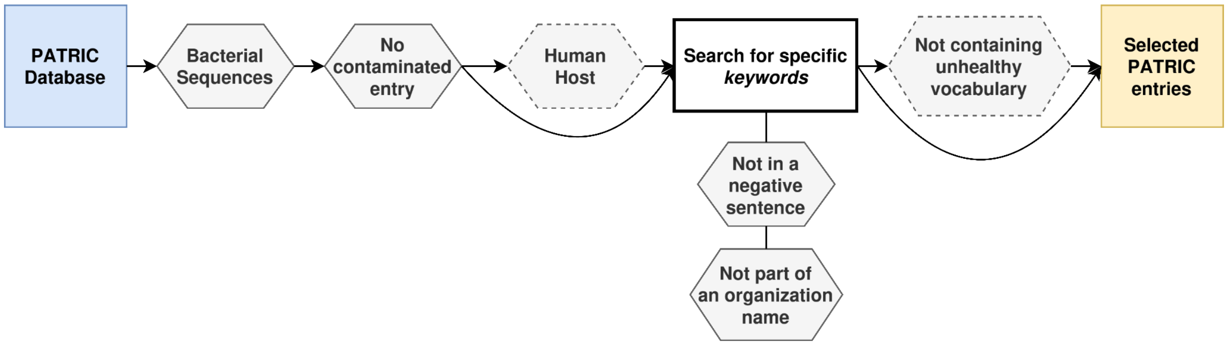

Figure S1: Scheme of selecting entries from the BV-BRC database.
Certain steps are skipped depending on the phenotype searched. Only entries for pathogenic capacity and "Non-pathogen" and "Microbiota" are required to have "Human host". Only entries for non-pathogens are required to "Not contain unhealthy vocabulary".

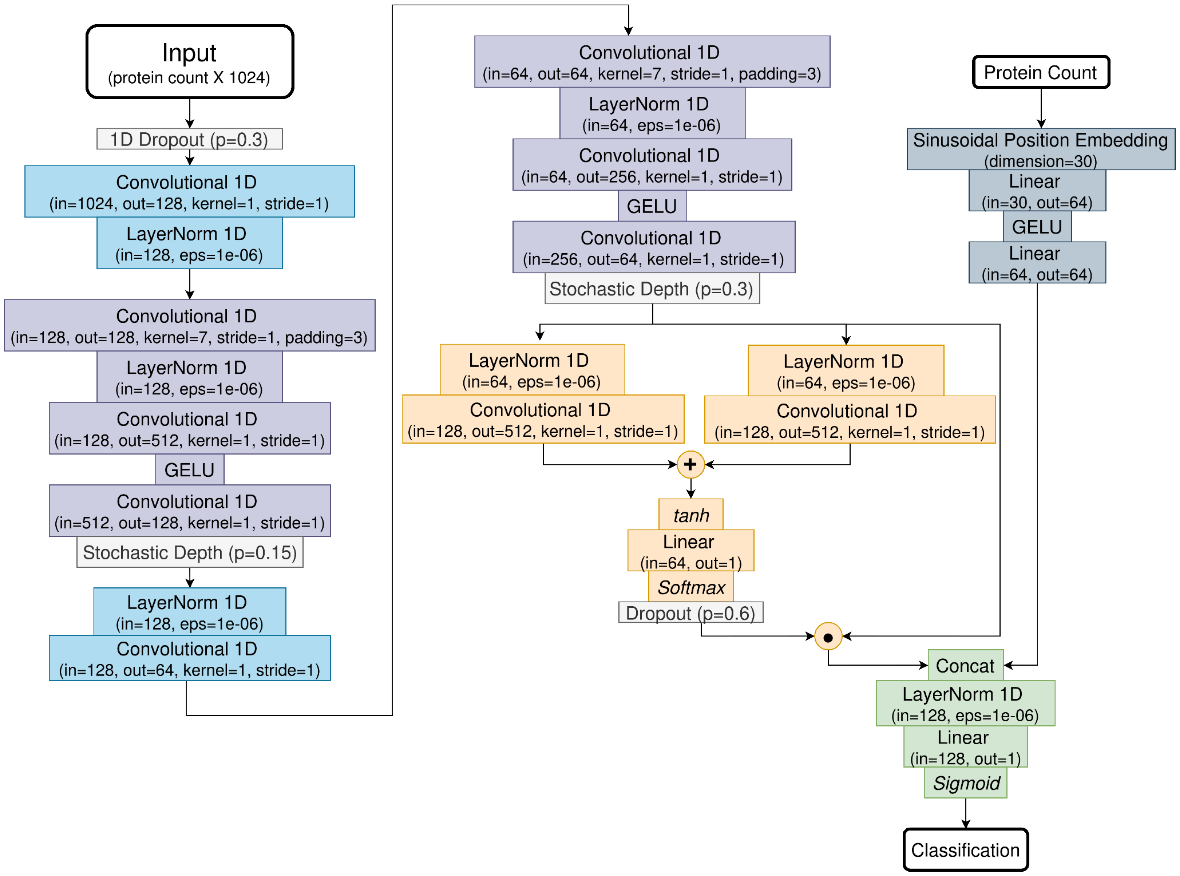

Figure S2: Technical scheme of PathogenFinder2 neural network.
The input (with shape *protein count X 1024*, which makes it of different shape depending on the genome), goes through a 1D dropout layer. This dropout layer drops protein positions with probability 0.3. Then it goes through a combination of Convolutional 1D and LayerNorm for 1D data that reduces the feature dimensions from 1024 to 128 (feature downsample, in blue). The next block is an adaptation of ConvNext to 1D data (in purple), followed by a stochastic depth layer for regularization. Next, another downsample feature block with a 1D Convolutional and LayerNorm for 1D data (blue). Afterwards, another ConvNext block (purple) followed by a stochastic depth layer. After all these layers, the

protein count dimension has kept its shape. Moreover, all the biases of the different layers were fixed to 0. This matrix is introduced in Bahdanau attention (in orange). The output of the attention layer is concatenated with the embedding of the protein count (grey), which goes through a feed-forward layer with a Softmax that is the output layer.

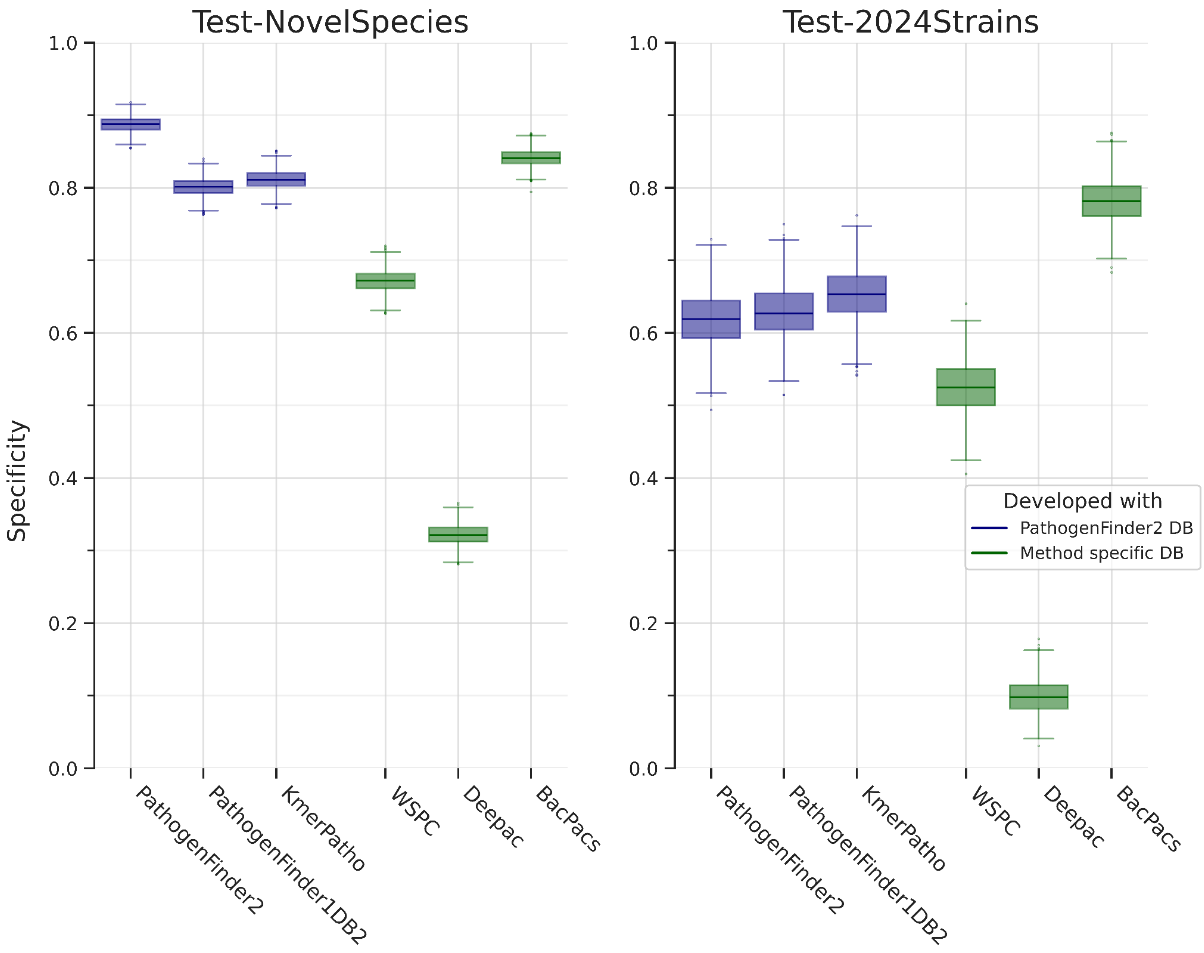

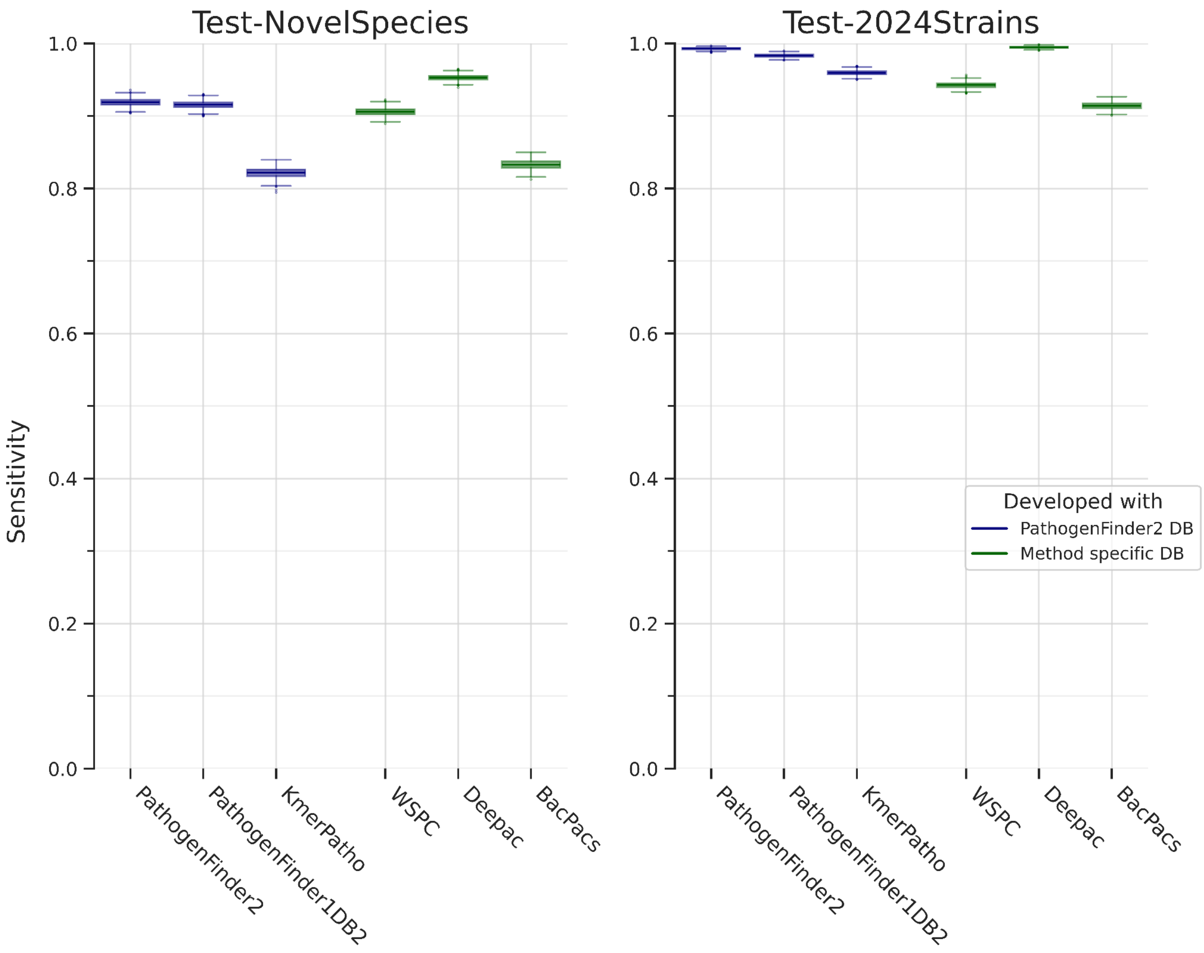

Figure S3: Specificity (top) and Sensitivity (bottom) on the Test-NovelSpecies and Test-2024Strains from the PathogenFinder2, previous state-of-the-art methods and baselines.

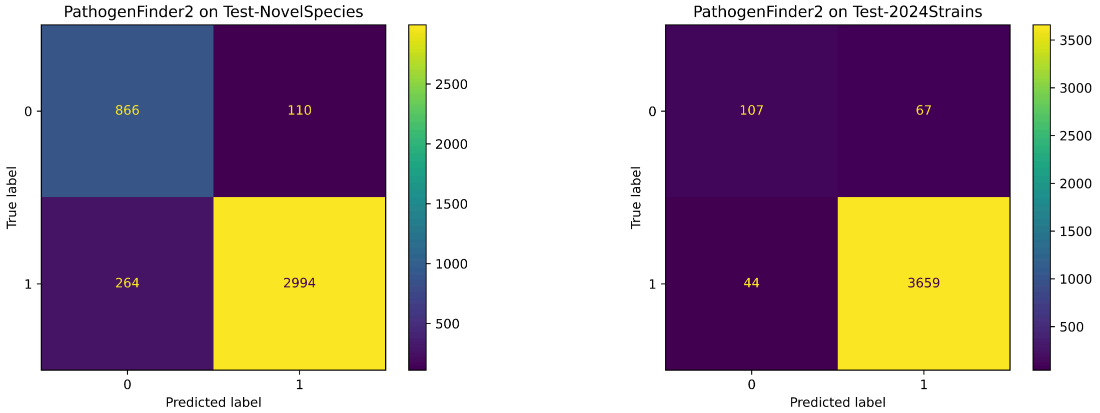

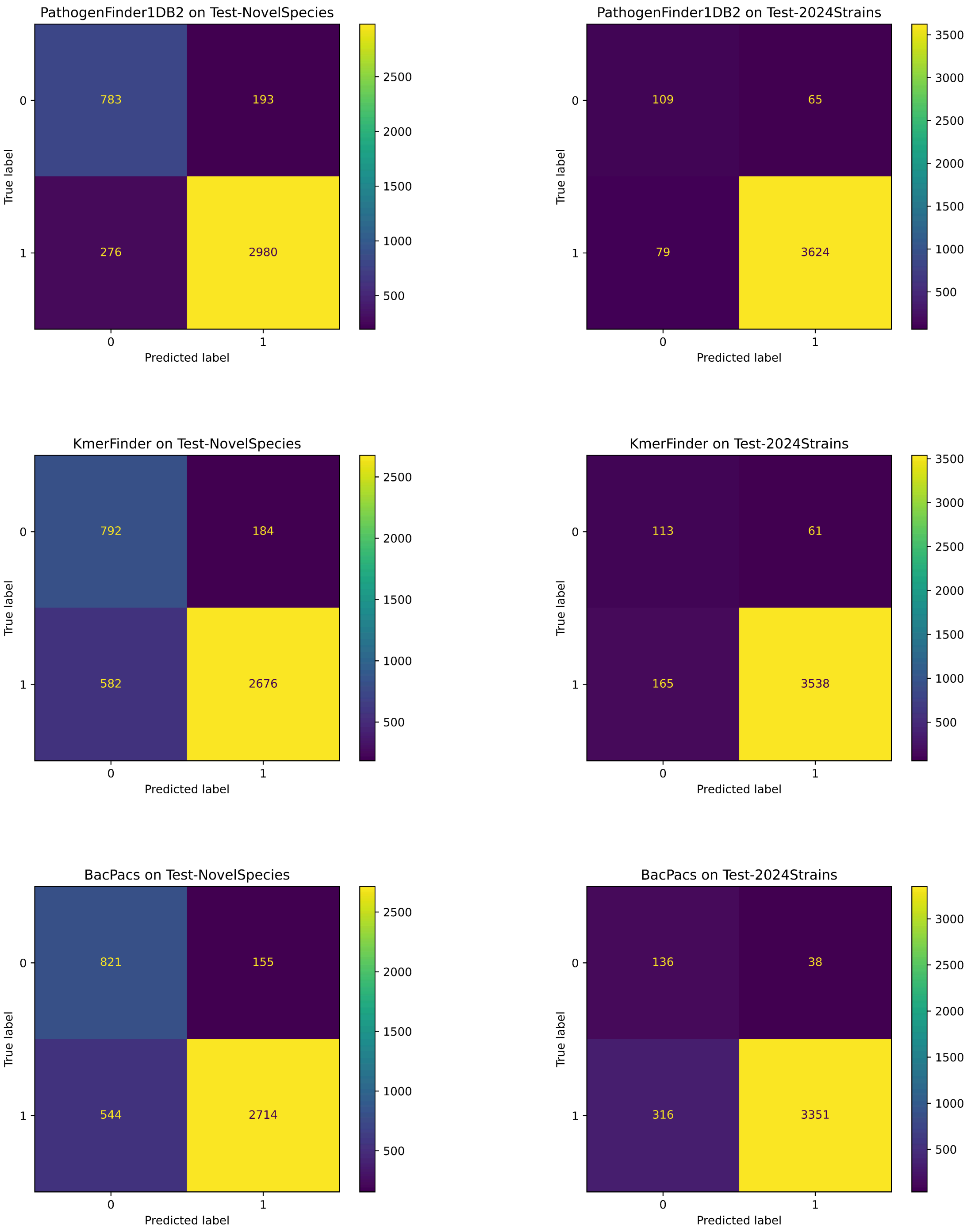
PathogenFinder1DB2 on Test-NovelSpecies
783
193
276
2980
PathogenFinder1DB2 on Test-2024Strains
109
65
79
3624
KmerFinder on Test-NovelSpecies
792
184
582
2676
KmerFinder on Test-2024Strains
113
61
165
3538
BacPacs on Test-NovelSpecies
821
155
544
2714
BacPacs on Test-2024Strains
136
38
316
3351
True label
Predicted label

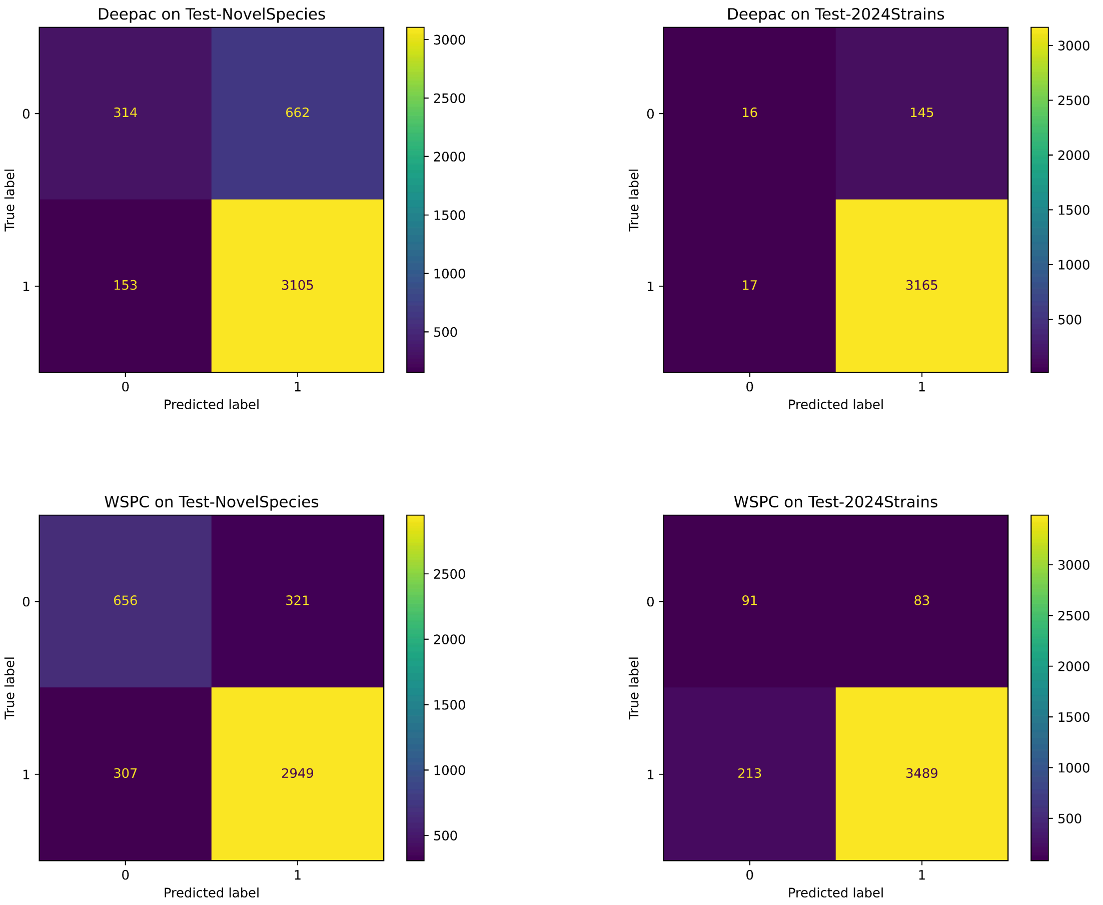

Figure S4. Confusion tables on the Test-NovelSpecies and Test-2024Strains from the PathogenFinder2, previous state-of-the-art methods and baselines.

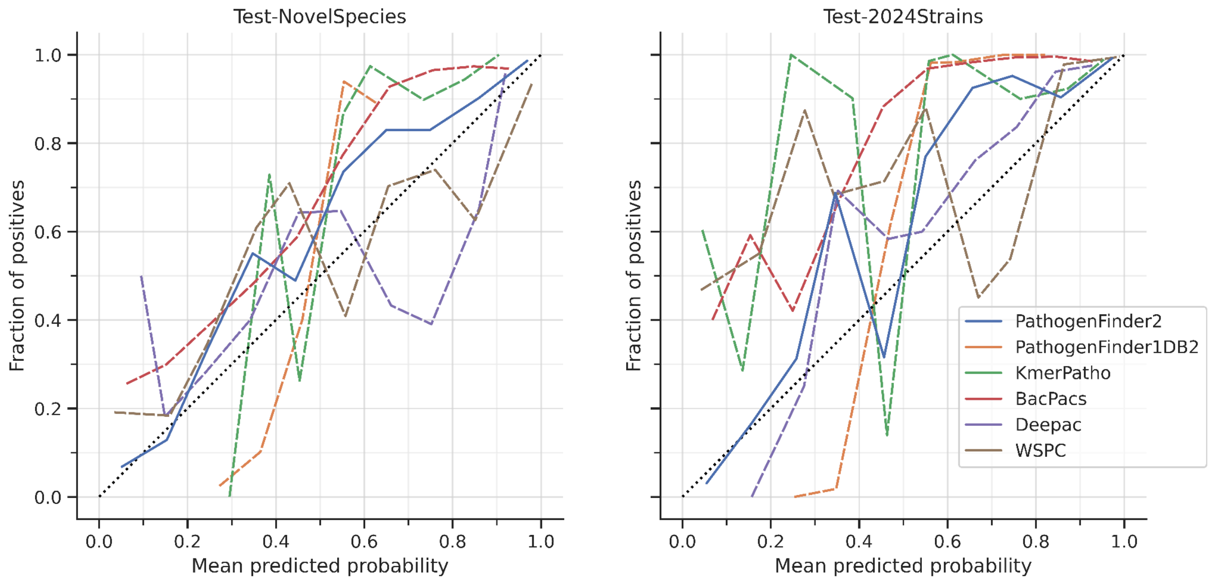

Figure S5. Calibration curves on the Test-NovelSpecies and Test-2024Strains from the PathogenFinder2, previous state-of-the-art methods and baselines.

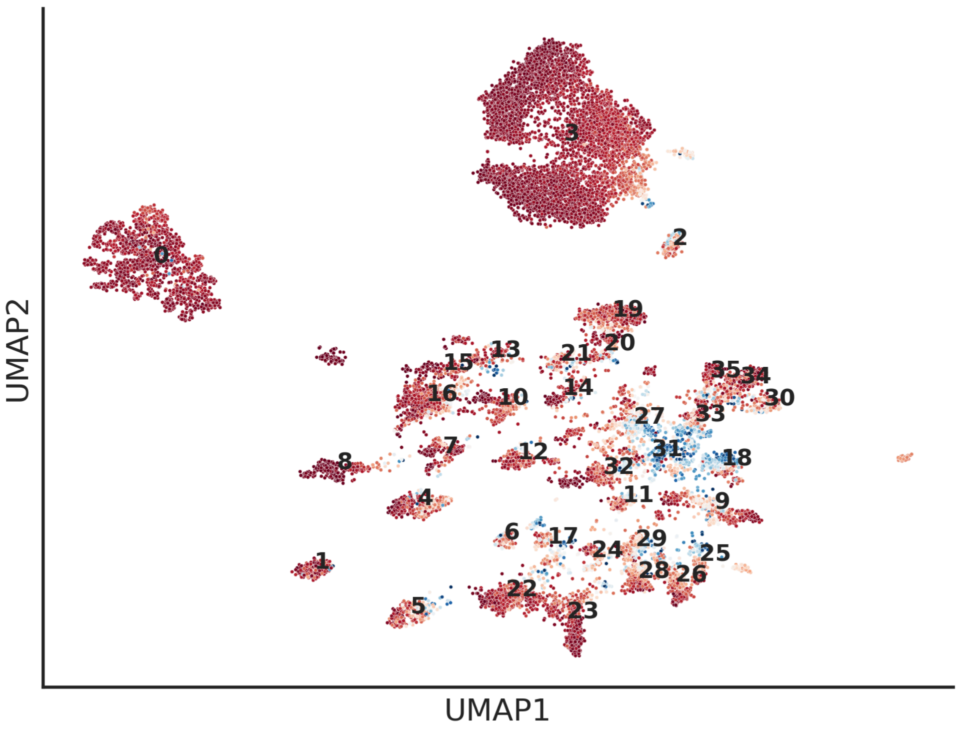

Figure S6: Predictions of PathogenFinder2 on the pathogenic subset of the PathogenFinder2 dataset.
The colors indicate the output of the sigmoid of the neural network model: from red (1, Pathogenic) to blue (0, Non-pathogenic). The UMAP is done on the embeddings produced by PathogenFinder2. The numbers indicate the clusters created with HDBScan, which can be mapped to the list of clusters in the Table S4, to map species on the *Bacterial Pathogenic Capacity Landscape*.

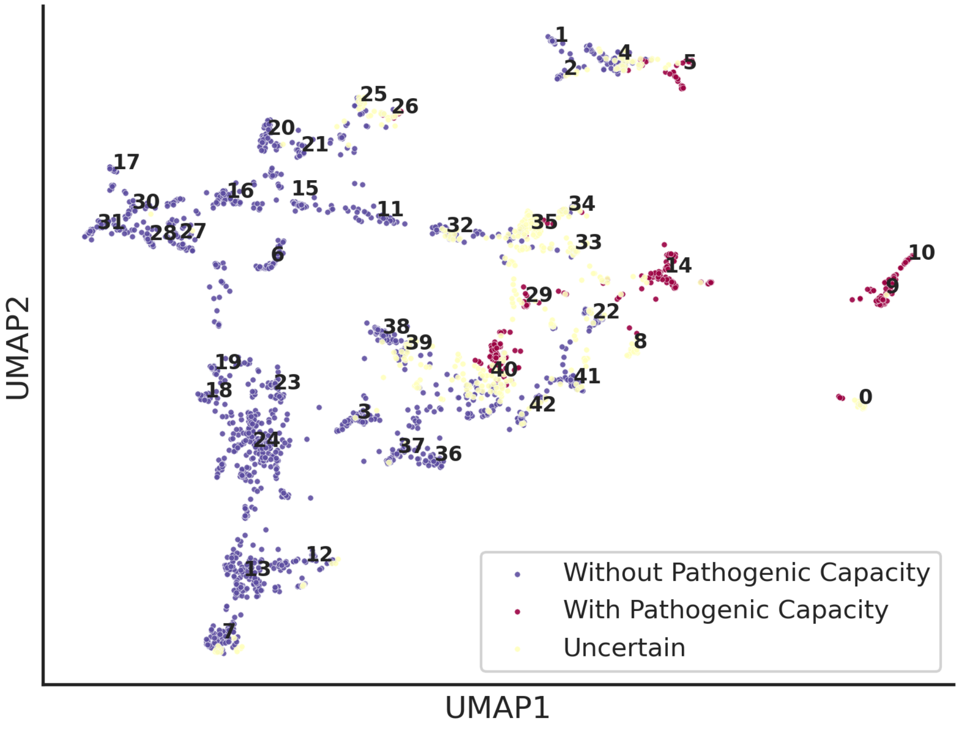

Figure S7: Distribution of PathogenFinder2 embeddings of the MAG sequences from (Jespersen *et al.*, 2023)
In color is the prediction done by the model. The numbers indicate the cluster that the sequences below belong to, done with HDBScan.

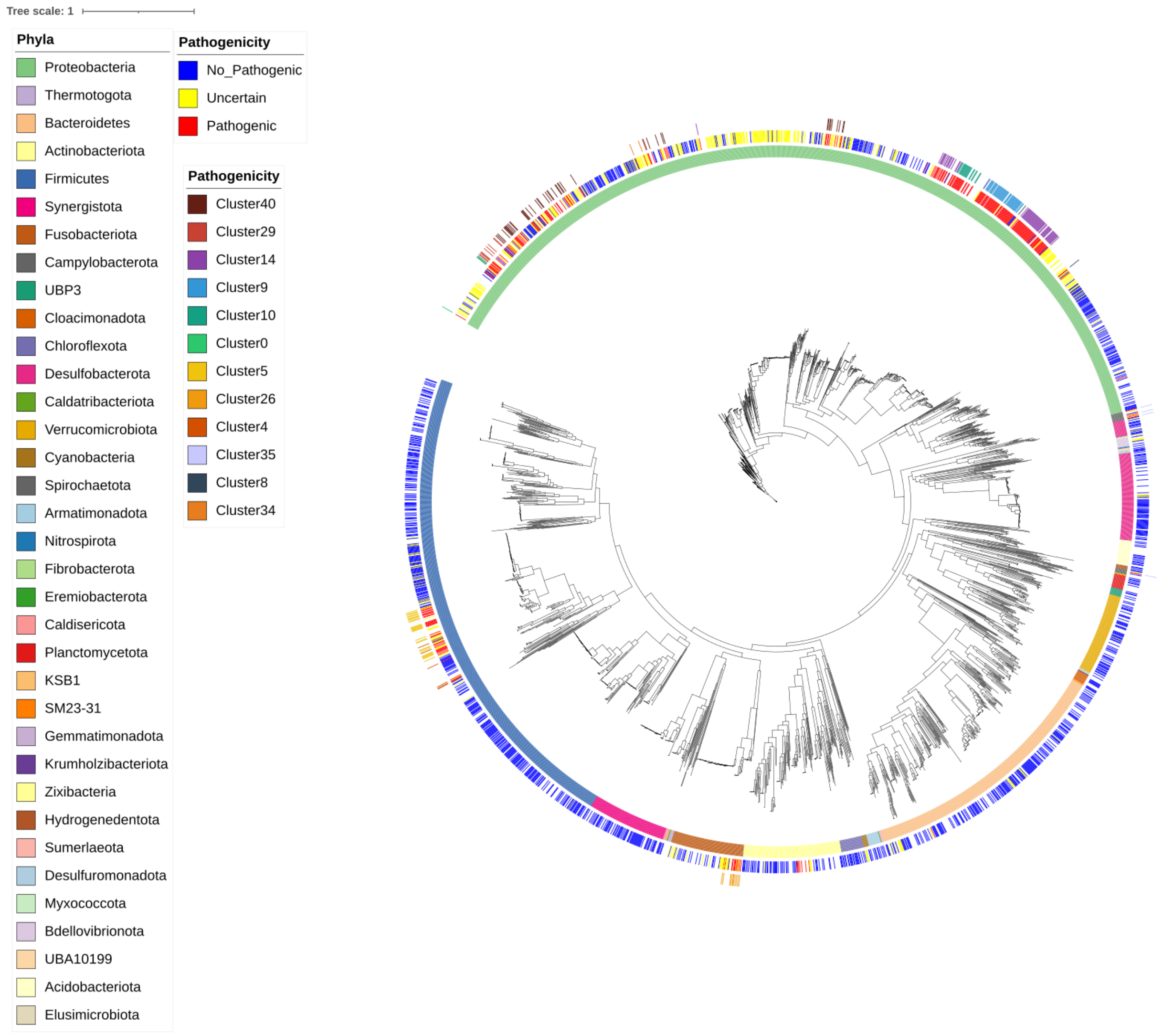

Figure S8: Taxonomy of the MAGs from (Jespersen *et al.*, 2023)**.**
The first circle is the phyla predicted by (Jespersen *et al.*, 2023)., the second the predictions of PathogenFinder2, while the third circle highlights clusters with pathogens indicated in Figure S7.

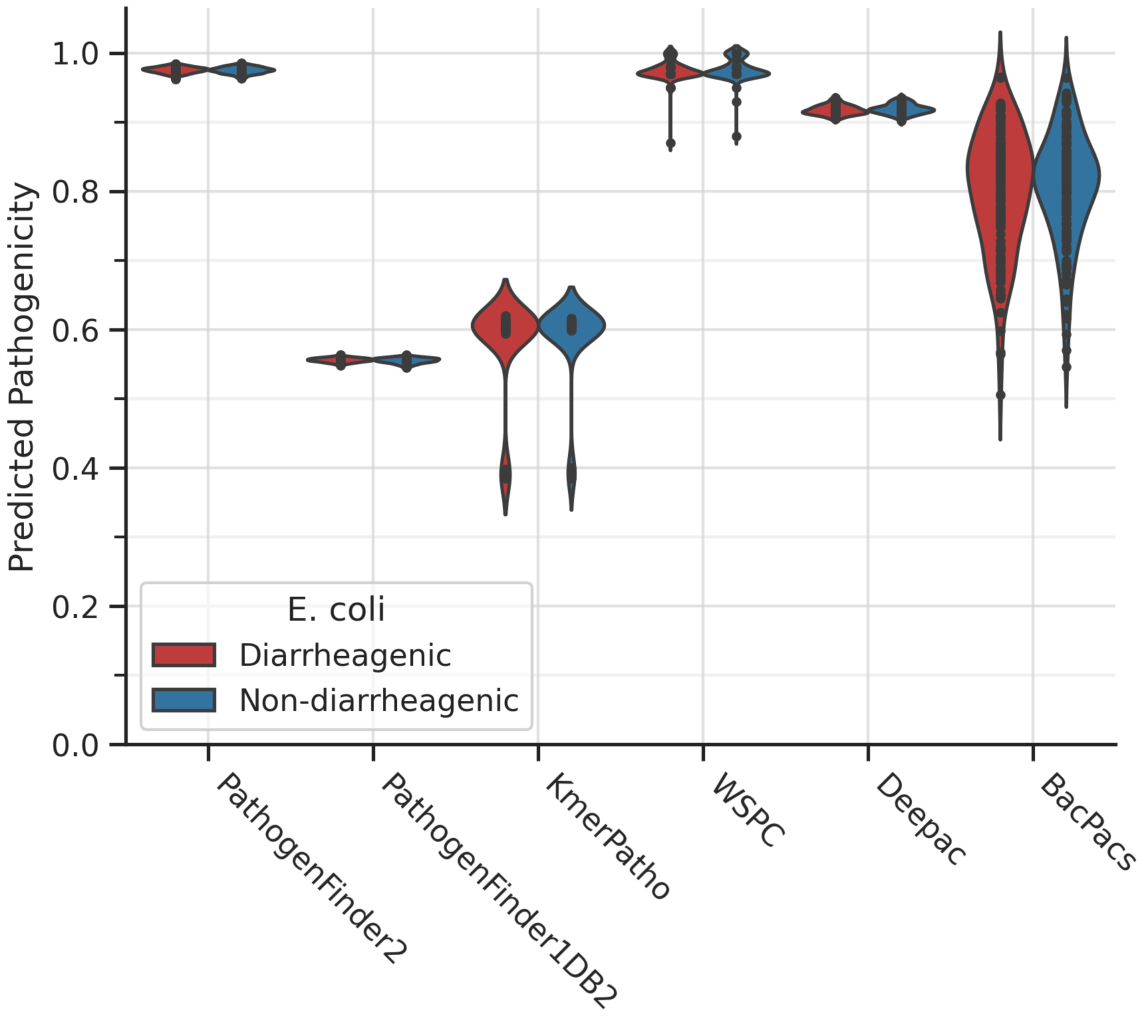

Figure S9: Predictions of the different pathogenic capacity predictors on the E. coli samples collected from diarrheagenic and non-diarrheagenic samples from (Hazen *et al.*, 2023).

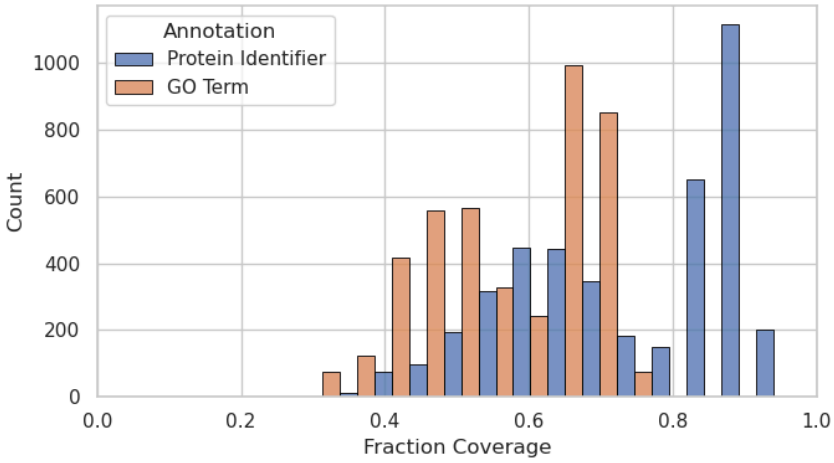
Annotation
Protein Identifier
GO Term
Count
Fraction Coverage

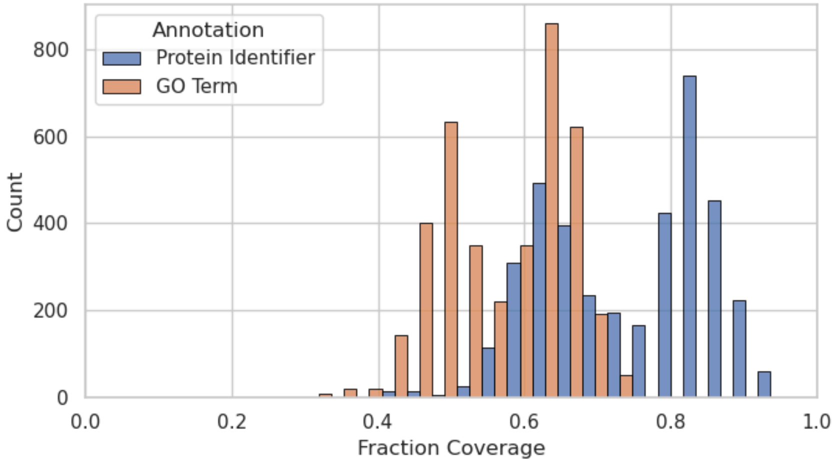
Annotation
Protein Identifier
GO Term
Count
Fraction Coverage

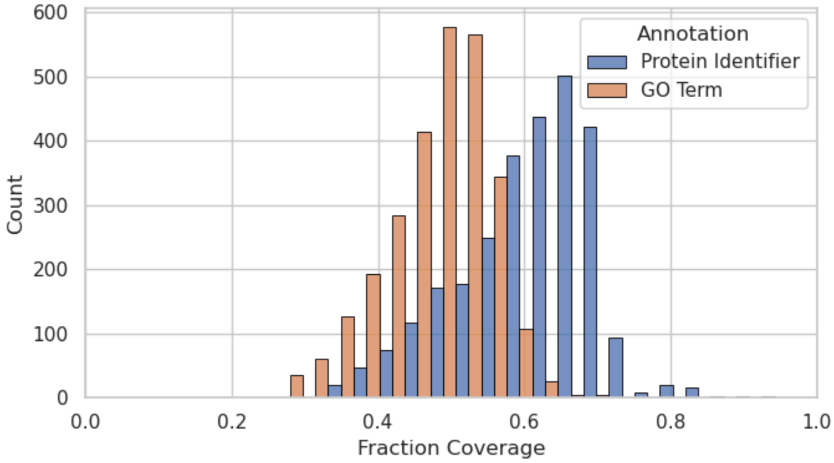
Annotation
Protein Identifier
GO Term
Count
Fraction Coverage

Figure S10: Coverage of the Protein Identifier and GO term of the proteomes in TestNovelSpecies (top), Test2024Strains (middle) and the mags from (Jespersen *et al.*, 2023) (bottom).

HyperParameter	Range Parameters	Chosen Parameters
Incorporate amount proteins	Concat, Add, False	Concat
Learning Rate	5e-05, 1e-04, 5e-04, 1e-03, 5e-03	5e-04
Sequential Dropout	0.4, 0.3, 0.2	0.3
Amount ConvNext Blocks	2, 3, 4	2
ConvNext Dimensions	64, 128, 256, 512	256
Stochastic Depth	0.1, 0.2, 0.3, 0.4	0.3
Attention Dimensions	64, 128, 256, 512	64
Attention Dropout	0.3, 0.4, 0.5, 0.6	0.6

Table S1: Hyperparameters selected through Hyperparameter optimization.

Protein Function	Protein (UniRef50 ID)
Mobility	**Flagellum:** A0A377CE65, UPI002021049C, A0A376J6R9, P24216, A0A2H9GEC1, A0A658Y519, A0A376MRW7, UPI00223FC8E7, A0A376Q7N2, P29744, A0A3S4IIY0. **Erythrocyte membrane binding protein:** A0A380E1C1
Adhesion	**Adhesin:** A0A2X1K134**,** A0A484X4U5, A0A376KQL1, A0A376KQL1, Q32DV6, A0AAE9PRL0, A0A376KTK2, A0A376KRZ5, A0A376DA64**. DUF1542 domain:** UPI0028DC8763**. Histidine triad domain:** A0A428HT79**. Fimbria:** A0A8T9CK35**,** UPI000AECBA48, A0A377ZUH8, A0A377WDJ6, A0A0C7K737, A0A377XIZ7. **VWFA;** A0AA37CTN4. **WxL:**A0A828QQA5**,**Q837W5,A0A829FD10, UPI0035A3D603, A0A829FF60, Q82ZI2, V7ZJ64, R2MC72. **VWA:**UPI002AB5021D**. Fibronectin:** A0A2X2YBB1**,** A0A9N8IGR9**. IG-like:** Q833X5, R3KJG5, A0A3F3NQX1
Barrier degradation	**N-acetylmuramoyl-L-alanine amidase:** A0A380EPW8**. Lipase:**A0A2X3CBG0**. Autolysin:** P06653**,** A0A2S1CXQ8. **Serralysin:** Q03023**. Endo-alpha-N-acetylgalactosaminidase:** Q8DR60**. beta-N-acetylhexosaminidase:** A0A4R3ZDQ6**. Hyaluronate lyase:**Q837L8
Biofilm	**Cellulose synthase:** P37650**,** A0A6L3Y0A4, A0A2X3CTY4, A0A8D6Q3D5 **Glucosyl transferase:** UPI00056B38CE, A0A943QTQ6
Anaerobic lifestyle	**Pyruvate formate-lyase**: A0A378A252. **Formate acetyltransferase**: A0A655BN26
Immune modulation	**Deubiquitination:** A0A3P5DT60
DNA	**Exoribonuclease**: A0A024L2P3. **Exodeoxyribonuclease**: P15032, A0A2X3F037, A0A377WIZ0, A0A4P0Y4I4, A0A8B3UZE9. **Exonuclease**: A0A377E4I2, A0A376W4B3, A0A376S820, A0A4T4CR35, A0A376PC22, A0A376W4B3. **Nuclease SbcCD**: A0AAE4DP17, UPI000D761D4D **Ribonucleoside triphosphate reductase**: A0A932ZA97, A0A2X3KGH4. **Anaerobic ribonucleoside reductase**: P28903, A0KHM1, A0A3S4IC06, A0A2X3KGH4. **DNA Ligase**: A0A6L3XYV6, A0A6L3XYV6.<br>**Others**: UPI002ED9F1EA.

Cell envelope	**Anchor protein:** A0A3S4NG01, A0A4U3KFC0**. Choline binding:** J7TSG7**. LPXTG:** A0A7T3VYA2**,** A0A2G0E6Y4, A0A930AN31. **Lipopolysaccharide:** P31554, A0A484VTJ8**. D-alanyl-D-alanine carboxypeptidase:** T0U2D3**. Septum formation:** A0A1L7CPP2**. GBS Bsp-like repeat-containing protein:** A0A943QU00**. Stalk domain-containing protein:** UPI0025CEC918. **MipA**: UPI0030EB7CB2, UPI003523C938, UPI0021C56F87, A0A7H4MK65. **Other**: YG1VC94, A0A377ZX21, UPI001CBDD196, UPI0002B55144, A0A3P5DS41. **Peptidoglycan glycosyltransferase:** F4NA87, A0A376GVY2
Secretion & toxins	**Autotransporter**: A0A1X3JJ14, Q32J02, P45508, A0A8S0G0G0, UPI0007B3DD72, UPI0009A527D6, UPI000459ED4E. **Secretion System (type VI)**: UPI0002C997FB,UPI002F3FDB27, R9RHK6, UPI00070728EE, F0KJ96. **HlyD**: UPI002FDFCD5E. **Rhs toxin**: UPI00111578E0, P16917, P16919, UPI00025CA3E5, UPI0013AF20F2. **ShET2**: A0AAN3NUG4, C3SDH0
Nutrient transport	**LCFA transporter:** A0A834ME79,A0AAP5MRU6. **Porin**: UPI0021CF0FDC, UPI002FF0DFE0, **OmpW**: A0A447RJP7. **Lactose permease:** N2A4B6. **ABC transporter:** K1J8L7.
Iron acquisition	**Deferrochelatase**: Q8XAS4, A0A2X1J1L9. **EfeUOB**: A0A2X3EIW7. **Hemin-degrading**: A0A484ZBD7, Q9HV89. **PvdL**: Q9I157. **Ferrichrome transporter:** A0A0C7KJM8. **HemS**: A0A919I0T4. **Ferrichrome receptor:** Q9I648. **FecR**:UPI0021564FC1. **Ferrioxamine receptor:** A0A378BUV2.
Carbon utilization	**Pullulanase**: UPI00291062F7, A0A4J1X9X5, Q8KLP1, A0A447S040. **Glycosyl hydrolase**: K1JCP5, A0A2X1MZ07,UPI0024955634, UPI002911E5A0, A0A3N6V9B5, P25718, UPI00352BF89C, UPI000A877F0A, UPI0023E440B1, UPI00036ACD60, Q833V2, UPI0030A8EE89, A0A377YW83
Metabolism	**Hydroxylamine reductase:** P75825. **5-formyltetrahydrofolate cyclo-ligase:** UPI001E5967B5. **Formate C-acetyltransferase:** UPI001C4F5A5B. **Glucose dehydrogenase:** W1DHM1, A0A3A1Y1R6, A0AA42MDJ3, A0A519EXL9, A0A4P0Y529, W9BGR5 A0A4U9HVX7. **Glucose-1-phosphatase:** A0A377ATJ5, A0A3E1ZXQ6. **Dextrasucrase**: Q55264, U2J3Q1. **Quinate dehydrogenase:** A0A377M186. **Cytochrome-c domain containing protein:** A0A3L0W265. **Hydrolase**: S7WUG3. **Amine oxidase**: A0A385EWU8, A0A377ZUN0, A0A377D2Z8, P46883.
Others	**FlxA-like protein**: Q32DE5. **Unknown**: A0A376FG28, P37681, P37342, UPI000A4D6E57, UPI000691E8B2. **Outer Membrane (lmp)**: A0A447RZ74, A0A2X3EA31.

Table S2: Selected UniRef and Uniprot IDs.
The highlighted proteins from genomes predicted to have pathogenic capacity in the Test-NovelSpecies and Test-2024Strains datasets were aligned against the UniRef50 database. The proteins are identified with UniProt and UniRef accession codes, as they appear in the UniRef50 database. The most frequent hits are listed in this table, along with their roles as virulence factors and in metabolic pathways.

Protein Identifier	GO term
• **Amino acid biosynthesis**: *Ornithine carbamoyltransferase*<br>• **Nucleotide biosynthesis**: *Dihydroorotase*<br>• **Carbohydrate metabolism**: *Alpha-amylase*<br>• **Energy production**: *Cytochrome c-552*<br>• **Degradative metabolism**: *Cellulase A*<br>• **Nutrient utilization***:* *Beta-glucosidase*<br>• **Peptide degradation***:* *Neutral endopeptidase*<br>• **Nutrient intake**: *TonB-dependent*<br>• **Protein synthesis**: *Glutamyl-tRNA(Gln) amidotransferase*<br>• **DNA mobility**: *Transposase; integrase, Tyrosine recombinase, Group II intron-encoded protein LtrA*<br>• **Biofilm formation***:* *Dextransucrase 1*<br>• **Adhesion**: *Autotransporter adhesin BtaE , Extracellular matrix-binding protein ebh*<br>• **Host-cell invasion**: *Internalins*<br>• **Host interaction***: Outer membrane protein SlpA*	• Iron ion transport (*Hemin transport protein HmuS*)<br>• DNA transposition* (*Transposase InsH for insertion sequence element IS5U)*<br>• Protein quality control for misfolded or incompletely synthesized proteins (*Lon protease*)<br>• Peptide transport (*Putative peptide-binding periplasmic protein*)<br>• Polysaccharide transport (*Fructooligosaccharide ABC transporter*<br>• N-acetylneuraminate catabolic process (*N-acetylmannosamine kinase*)<br>• Iron-sulfur cluster assembly (*Iron-sulfur cluster assembly protein SufD*)<br>• Cellulose catabolic process (*Beta-D-glucoside glucohydrolase*)<br>• Maltodextrin transmembrane transport (*Putative binding protein BAB2_0491*)<br>• Menaquinone biosynthetic process (*SEPHCHC synthase*)

receptor<br>• **Membrane transport***:* *Outer membrane transporter CdiB-2, Dipeptide-binding protein, Hemin import ATP-binding protein, PTS system glucose-specific EIICBA component*	*substrate-binding protein*)<br>• L-tryptophan biosynthetic process (*N-(5'-phosphoribosyl)anthranilate isomerase*)<br>• Glucan catabolic process (*Beta-D-glucoside glucohydrolase*)<br>• Intracellular protein transmembrane transport (*Protein translocase subunit SecA*)	• Chorismate biosynthetic process (*3-phosphoshikimate 1-carboxyvinyltransferase*)<br>• Establishment of competence for transformation (*ComE operon protein 3*)

Table S3: Protein identifiers and functions highlighted by attention scores using preranked GSEA on bacteria predicted without pathogenic capacity.
Table with lists of the Protein Identifiers and GO terms that GSEA significantly positively enriched (FDR q-value <0.01) by attention scores on bacteria predicted without pathogenic capacity in the sets Test-NovelSpecies and Test-2024Strains. The names of the Protein Identifiers are organized in protein functions, of which some coincide to be virulence factors categories commonly described. Their order of appearance on the lists is not related to the degree of enrichment. Uncharacterized proteins confidently enriched by GSEA were excluded. The GO terms are listed by their median Normalized Enrichment Score (NES); if a GO term appeared in both test sets (marked with an asterisk), only the one with highest median NES is shown.

Cluster Number	Most common Species
0	*Helicobacter pylori*
1	*Campylobacter jejuni, Campylobacter coli*
2	*Providencia alcalifaciens, Proteus mirabilis*
3	*Escherichia coli, Klebsiella pneumoniae, Enterobacter hormaechei, Klebsiella variicola, Enterobacter cloacae, Citrobacter freundii, Salmonella enterica*
4	*Burkholderia cepacia, Burkholderia pseudomallei*
5	*Haemophilus influenzae, Haemophilus haemolyticus*
6	*Streptococcus mutans*
7	*Mycobacteroides abscessus, Mycobacterium avium*
8	*Streptococcus pneumoniae*
9	*Clostridioides difficile, Clostridium perfringens*
10	*Vibrio parahaemolyticus, Vibrio cholerae*
11	*Campylobacter concisus*
12	*Neisseria meningitidis*
13	*Leptospira interrogans, Legionella pneumophila*
14	*Stenotrophomonas maltophilia, Achromobacter xylosoxidans*

15	*Acinetobacter junii, Acinetobacter haemolyticus*
16	*Acinetobacter baumannii, Acinetobacter bereziniae*
17	*Streptococcus salivarius, Streptococcus sobrinus, Streptococcus sanguini*
18	*Clostridium sp., Clostridium perfringens*
19	*Pseudomonas aeruginosa*
20	*Pseudomonas aeruginosa, Pseudomonas putida*
21	*Aeromonas veronii, Aeromonas hydrophila, Aeromonas caviae*
22	*Streptococcus oralis, Streptococcus mitis, Streptococcus sanguinis*
23	*Streptococcus pyogenes, Streptococcus parasanguinis, Streptococcus anginosus*
24	*Staphylococcus aureus, Staphylococcus pseudintermedius*
25	*Listeria monocytogenes, Enterococcus casseliflavus*
26	*Enterococcus faecium*
27	*Gardnerella vaginalis*
28	*Enterococcus faecalis*
29	*Staphylococcus epidermidis, Staphylococcus haemolyticus*
30	*Bacteroides uniformis*
31	*Lactobacillus spp., Clostridium spp., Neisseria spp.*
32	*Corynebacterium spp.*
33	*Porphyromonas gingivalis, Bacteroides stercoris*
34	*Bacteroides spp.*
35	*Bacteroides fragilis*

Table S4: Main bacteria species on each of the clusters shown in the Figure S6

References

Hazen,T.H. *et al.* (2023) Genomic diversity of non-diarrheagenic fecal Escherichia coli from children in sub-Saharan Africa and south Asia and their relatedness to diarrheagenic E. coli. *Nat. Commun.*, **14**, 1400.

Jespersen,M.L. *et al.* (2023) Global within-species phylogenetics of sewage microbes suggest that local adaptation shapes geographical bacterial clustering. *Commun. Biol.*, **6**, 1–9.
